# Epsilon-Fe_2_O_3_ is a novel intermediate for magnetite biosynthesis in magnetotactic bacteria

**DOI:** 10.1186/s40824-019-0162-1

**Published:** 2019-08-02

**Authors:** Tong Wen, Yunpeng Zhang, Yuanyuan Geng, Junquan Liu, Abdul Basit, Jiesheng Tian, Ying Li, Jilun Li, Jing Ju, Wei Jiang

**Affiliations:** 1 State Key Laboratory of Agrobiotechnology and Ministry of Agriculture Key Laboratory of Soil Microbiology, College of Biological Sciences, China Agricultural University, Beijing, 100193 People’s Republic of China; 2 College of Chemistry and Molecular Engineering, Peking University, Beijing, 100871 People’s Republic of China; 3 Department of Biology Science and Technology, Baotou Teacher’s College, Baotou, 014030 People’s Republic of China; 4 Agricultural Utilization Research Center, Nutrition and Health Research Institute, COFCO Corporation, Beijing, 102209 People’s Republic of China

**Keywords:** *ε*-Fe_2_O_3_, Magnetosome maturation, *Magnetospirillum gryphiswaldense* MSR-1, Time course experiment

## Abstract

**Background:**

Natural biological magnetite nanoparticles are widely distributed from microorganisms to humans. It is found to be very important in organisms, especially in navigation. Moreover, purified magnetite nanoparticles also have potential applications in bioengineering and biomedicine. Magnetotactic bacteria (MTB) is considered one of the most abundant species around the world which can form intracellular membrane enveloped magnetic nanoparticles, referred to as magnetosomes. To our knowledge, the biomineralization of magnetosome in MTB involves a serious of genes located on a large unstable genomic region named magnetosome island, which specially exists in MTB. The magnetite core of magnetosome formed via a Fe (III) ion intermediates, for instance, *α*-Fe_2_O_3_ and ferrihydrite. Though the biosynthesis of magnetosome represents a general biomineralization mechanism of biogenic magnetite, knowledge of magnetosome biosynthesis and biomineralization remains very limited.

**Method:**

Cells used in this study were cultured in a 7.5-L bioreactor, samples for intermediate capture were taken each certain time interval after the generation of magnetosome biosynthesis condition. High-resolution transmission electron microscopy were used to analyze the detailed structure of magnetosomes. The parameters of the crystal structures were obtained by Fast Fourier Transform analyses.

**Results:**

In this study, we identified a novel intermediate phase, *ε*-Fe_2_O_3_, during the magnetite maturation process in MTB via kinetic analysis. Unlike *α*-Fe_2_O_3_, which has been reported as a precursor during magnetosome biosynthesis in MTB before, *ε*-Fe_2_O_3_, due to its thermal instability, is a rare phase with scarce natural abundance. This finding confirmed that *ε*-Fe_2_O_3_ is an important novel intermediate during the biomineralization of magnetosome in MTB, and shed new light on the magnetosome biosynthesis pathway.

**Electronic supplementary material:**

The online version of this article (10.1186/s40824-019-0162-1) contains supplementary material, which is available to authorized users.

## Introduction

Magnetite nano-particles are widely distributed mineral compounds found in various organisms including bacteria, bees, pigeons or even in human beings [1–4]. Membrane-enveloped magnetite, biosynthesized by magnetotactic bacteria (MTB), is referred to as magnetosomes [5, 6]. Although magnetosomes have great potential in bioengineering and biomedicine applications [7–9], the intracellular biomineralization mechanism of magnetosomes remains poorly understood. Since the discovery of MTB, it has been believed to be an optimal material for the study of biogenic magnetite biomineralization because of the simple structure of these organisms.

In most MTB, the magnetosomes are composed of magnetic magnetite (Fe_3_O_4_) or sulfide greigite (Fe_3_S_4_) [10, 11] enveloped by biological membrane [12]. Under the action of a skeleton-like protein MamK and its binding partner MamJ, single magnetosome are arranged in chains parallel to the long axis of the cell [13–15].

Many studies using genetic, biochemical and physicochemical approaches have been performed to reveal the biological control of magnetosome synthesis in MTB. A series of genes were identified to be involved in this process, the *mam* genes, comprising the magnetosome island (MAI), which is a large unstable genomic region spanning 80–150 kb in length in different MTBs [16, 17]. However, knowledge about the chemical route of iron during the magnetosome formation process is limited and how iron ions are incorporated into magnetite is still a matter of debate. Previous studies using Mőssbauer spectroscopy suggested that ferrihydrite is a precursor for magnetite formation [18, 19]. This was further confirmed in *Magnetospirillum gryphiswaldense* MSR-1, a type strain for MTB study, using Fe K-edge X-ray absorption near edge structure (XANES) and high-resolution transmission electron microscopy (HRTEM) analysis [20]. Real-time study about magnetosome biosynthesis using transmission electron microscopy (TEM) and X-ray absorption spectroscopy in MSR-1 revealed that full-sized magnetosomes formed within 15 min and immature magnetosomes contain a surface layer of hematite phase [21]. In *mamXY* gene cluster mutants of MSR-1, distinct types of *α*-Fe_2_O_3_ particles co-existed with magnetite, hinting that the transformation of hematite phase to magnetite phase is a biocatalysis process [22]. Magnetite formation from a phosphate-rich ferric hydroxide via nanometric ferric (oxyhydr) oxide intermediates was recently shown in *Magnetospirillum magneticum* AMB-1 [23]. Together, these studies showed that the formation of magnetite in organisms possibly occured via Fe (III) ion intermediates and their oxides, hydroxides or oxyhydroxides.

It was suggested in the previous study that a thermal unstable phase of ferric oxide, *ε*-Fe_2_O_3_, was found in a mutant of MSR-1, which raised the possibility of a new type of intermediate during biomineralization process [24]. In the present study, a kinetic analysis was performed to study the magnetosome maturation process from the early stage of its formation. The results showed that at each time point during maturation of magnetosomes after induction, magnetite co-existed with various iron-containing phases, including *α*-Fe_2_O_3_, *ε*-Fe_2_O_3_ and other undefined phases. This result indicated that mature magnetosomes form via different types of iron-containing intermediates, including some rare phases such as *ε*-Fe_2_O_3_.

## Materials and methods

### Bacteria and growth conditions

*M. gryphiswaldense* MSR-1 was cultured in SLM at 30 °C as described previously [25]. The medium contained (per liter double distilled water) 2.5 g sodium lactate, 0.4 g NH_4_Cl, 0.1 g yeast extract, 0.5 g K_2_HPO_4_, 0.1 g MgSO_4_ • 7H_2_O, 0.05 g sodium thioglycolate and 5 mL trace element mixture. The strains were cultured at 30 °C in 250-mL serum bottles containing 100 mL medium with shaking at 100 rpm. Antibiotics nalidixic acid (Nx) was applied at 5 μg/mL for MSR-1.

### Cell magnetic response curve of MSR-1 strain

The coefficient of magnetism (Cmag) value of MSR-1 was calculated from measurements of the maximal and minimal optical density (OD_600_) using a UV-visible spectrophotometer (UNICO2100; UNICO Instrument Co., Shanghai, China) [26].

### Transmission electron microscope measurement

The structural details of the nanoparticles in MSR-1 samples from different time interval were analyzed by the HRTEM method using a JEM-2100F, which was operated at 200 kV, and it was equipped with a field emission gun, ultra-high-resolution pole piece, and ultrathin window JEOL detector. HRTEM images were obtained with an OSIS CANTEGA CCD camera. The crystals’ structural parameters were obtained by Fast Fourier Transform (FFT) analyses.

### Time-course experiment for the detection of the intermediate phase at the early stage of magnetosome formation

MSR-1 cells were cultured in 5.0 L of SLM medium in a 7.5-L bioreactor as described previously [27], until the relative dissolved oxygen concentration (DO) decreased to 1%. Then, 30 mL of 10 mM ferric citrate was added to the culture to induce the synthesis of magnetosomes. Samples for HRTEM observation were collected at 0, 5, 10, 15 and 25 min after induction and fixed immediately with 2.5% glutaraldehyde. After washing three times with double-distilled water, the cells were dispersed onto a copper grid for HRTEM analysis.

## Results

**Fig. 1:**
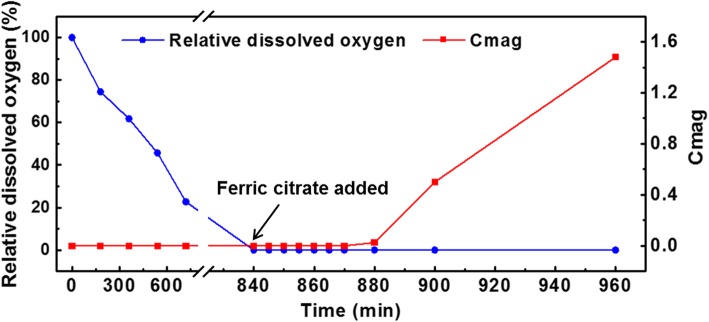
Time dependence of relative dissolved oxygen and magnetic response (Cmag) of MSR-1 cultured in the SLM media. 60 μM ferric citrate was added when the relative dissolved oxygen decreased to 1% in the culture to induce the formation of magnetosome. Then samples were taken for certain interval after the inducing of ferric citrate. Thereafter they were collected for HRTEM observation

**Fig. 2:**
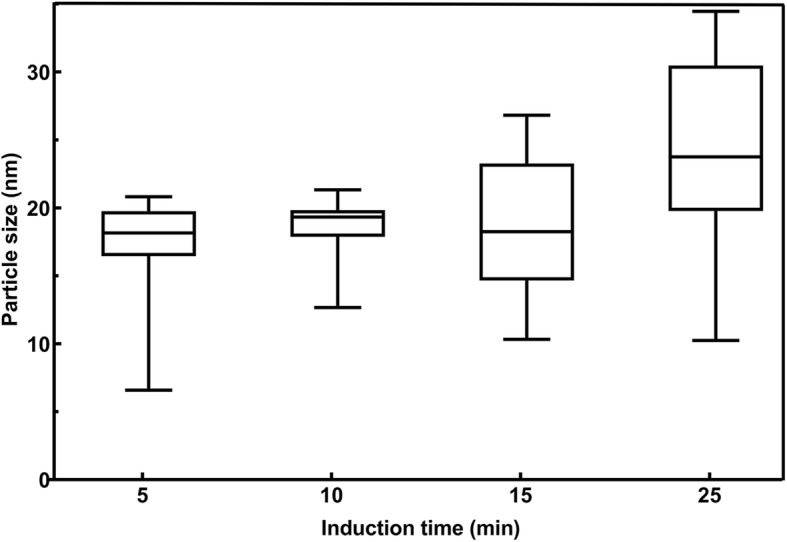
The size distribution of magnetosomes at different time interval after the induction of ferric citrate

**Fig. 3:**
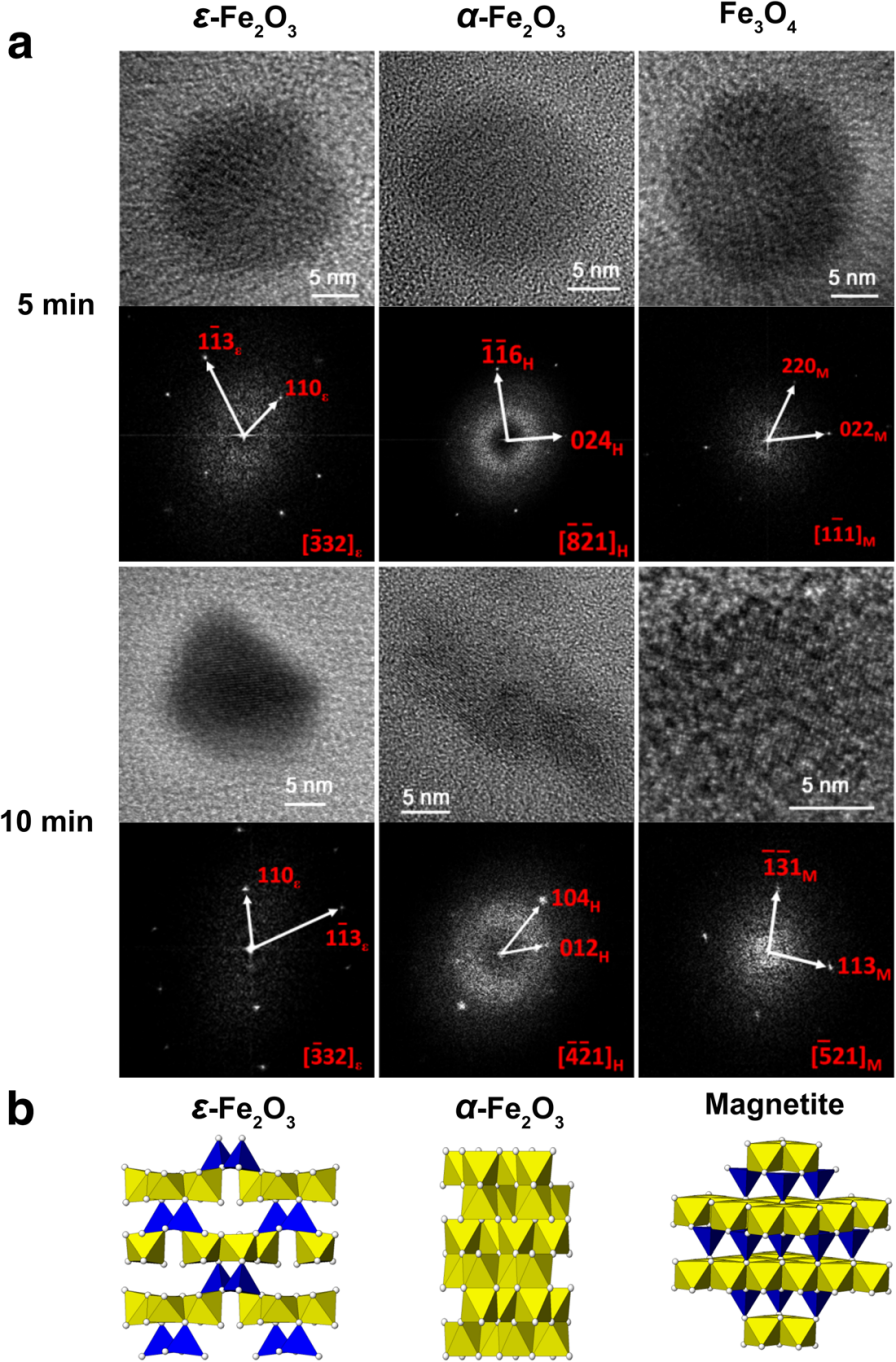
Component analyses of intracellular iron oxide nanoparticles in MSR-1 wild type after the induction of ferric citrate for 5 min and 10 min by HRTEM**. a**. HRTEM analysis of intracellular iron oxide nanoparticles form samples, FFT analyses are shown below the corresponding HRTEM images. *M*, *ε*, and *H* subscripts shown in FFT pattern denote Fe_3_O_4_ (magnetite), *ε*-Fe_2_O_3_, and *α*-Fe_2_O_3_ (hematite) phases, respectively. The time shown beside the individual HRTEM image is the interval when the samples were taken out after the induction of ferric citrate. **b**. The schematic representation of crystal structures for magnetite, hematite and *ε*-Fe_2_O_3_

**Fig. 4:**
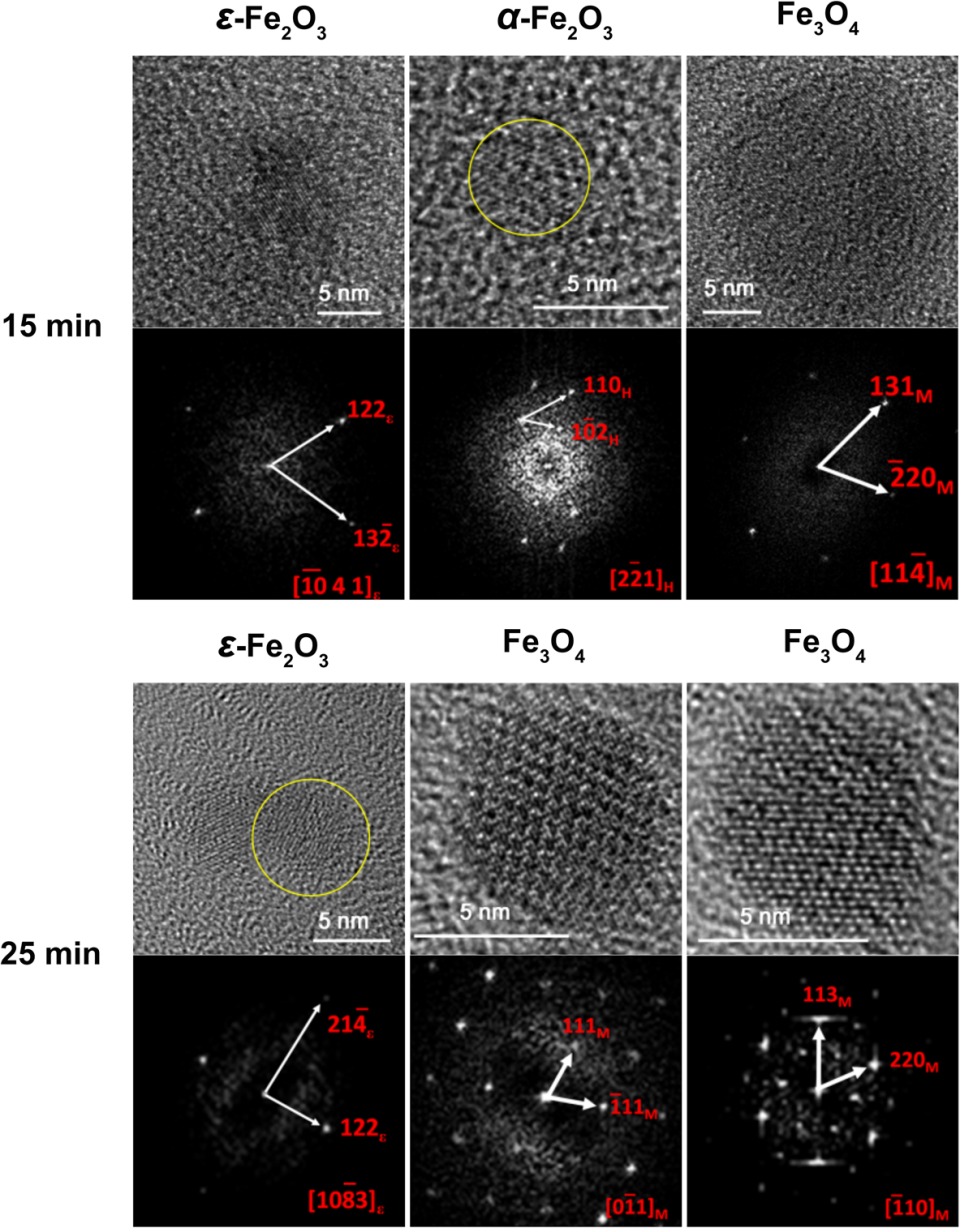
Component analyses of intracellular iron oxide nanoparticles in MSR-1 wild type after the induction of ferric citrate for 15 min and 25 min by HRTEM. HRTEM analysis of intracellular iron oxide nanoparticles form samples, FFT analyses are shown below the corresponding HRTEM images. *M*, *ε*, and *H* subscripts shown in FFT pattern denote Fe_3_O_4_ (magnetite), *ε*-Fe_2_O_3_, and *α*-Fe_2_O_3_ (hematite) phases, respectively. The time shown beside the individual HRTEM image is the interval when the samples were taken out after the induction of ferric citrate. Yellow cycles are for eye guide

**Fig. 5:**
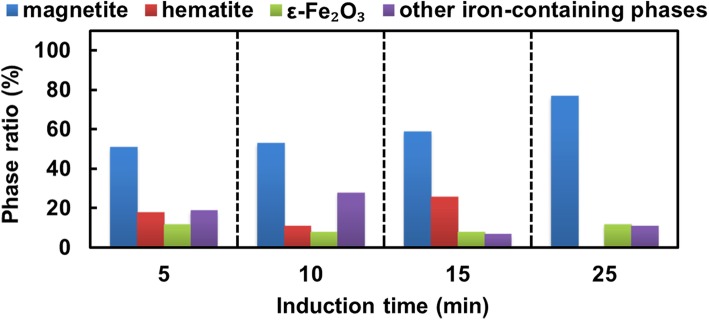
Phase ratio of various iron containing nanoparticles synthesized by MSR-1 at each time interval after the inducing of magnetosome formation. The results are based on HRTEM analyses and showed that at the early stage of magnetosome formation, multiple iron oxide nanoparticles can co-exist in the cells besides magnetite, including hematite, *ε*-Fe_2_O_3_ and other iron containing phases. Some representative HRTEM images and their FFT analyses are listed in SI

For this study, in order to initiate the biomineralization of large amount of magnetite accurately, cultivation of *M. gryphiswaldense* MSR-1 was carried out in a 7.5-L bioreactor. After the relative dissolved oxygen decreased to 1%, ferric citrate was added to induce magnetosome biosynthesis, samples were taken at different time points and fixed in 2.5% glutaraldehyde to maintain their original state (Fig. 1). Then, cells were prepared for HRTEM analysis. From the result, even at the 0-min interval, the formation of several iron-containing nanoparticles was evident, though the size of the particles are very small. Then, the average diameter of magnetosomes grows with time passed (Fig. 2). During this time, the predominant phase composing the particles was magnetite, but other iron-containing phases, including *α*-Fe_2_O_3_ and *ε*-Fe_2_O_3_, were present (Fig. 3a and Fig. 4, the data of *ε*-Fe_3_O_3_ measured in this work and the theoretical data of *ε*-Fe_2_O_3_ and Fe_3_O_4_ (magnetite) are listed in Additional file 1: Table S1). The finding of *α*-Fe_2_O_3_ as an intermediate in magnetite biomineralization is in accordance with previously reports, but the existence of *ε*-Fe_2_O_3_, which is an unstable phase under normal conditions, has never been reported before. For this assay, a total of more than 400 particles were analyzed, and Fig. 5 shows the phase ratio of different iron species by induction time point, Fe_3_O_4_ (magnetite), *ε*-Fe_2_O_3_, and *α*-Fe_2_O_3_ (hematite) phases are denoted by subscripted *M*, *ε*, and *H*, respectively (Some of the data measured in this assay are listed in Additional file 1: Figure S1). At each induction time point before the formation of mature magnetosomes, iron oxides always co-exist and proportion of magnetite increased with time passed. This result indicates that various iron-containing precursors co-exist as intermediate phases at the initiation phase of magnetosome biomineralization. The HRTEM data are shown in Fig. 3a, Fig. 4 and the original images with high resolution are available in Additional file 1: Fig. S2. Other iron-containing phases will be discussed in detail in another paper.

## Discussion

Recent studies have confirmed the existence of *α*-Fe_2_O_3_ at the early stage of magnetosome formation and act as a precursor phase [20, 21, 23]. In this study, we discovered that a thermodynamically unstable iron oxide phase, *ε*-Fe_2_O_3_, co-existed with *α*-Fe_2_O_3_ at the early stage of magnetosome synthesis, thus demonstrating that both of these two iron oxide phases are intermediates during the maturation of magnetosomes.

*α*-Fe_2_O_3_, *γ*-Fe_2_O_3_, and Fe_3_O_4_ are the most frequent iron oxides present in bulk in nature (crystal structures of Fe_3_O_4_ and *α*-Fe_2_O_3_ are shown in Fig. 3b) [28]. By contrast, *ε*-Fe_2_O_3_ is a rare phase with scarce natural abundance due to its thermal instability [29, 30]. Recently, the natural occurrence of a *ε*-Fe_2_O_3_ phase in some plants has been reported [31]. The crystal structure of the *ε*-Fe_2_O_3_ phase is an orthorhombic non-centrosymmetric structure with Fe atoms occupying four distinct nonequivalent crystallographic sites, including one tetrahedral site and three different octahedral sites (Fig. 3b) [32]. In the context of nano-materials, this structure is interesting, perhaps indicating a critical role of high surface energy, a characteristic of most nanostructures, in the formation of the epsilon phase with diameters of approximately 20 nm.

Similarities between the crystal structures of magnetite and *ε*-Fe_2_O_3_ are shown in Fig. 3b. Both tetrahedral and octahedral coordination of Fe and O occur in the two iron oxides; the stacking pattern of the two is formed by octahedral layers alternating with tetrahedral layers, with the exception of ordered octahedral vacancies in *ε*-Fe_2_O_3_. These similarities facilitate the structural transformation of *ε*-Fe_2_O_3_ to magnetite. Therefore, the formation of magnetite-containing magnetosomes via a *ε*-Fe_2_O_3_ intermediate may be more facile, even though *α*-Fe_2_O_3_ is more thermally stable.

## Conclusions

**Fig. 6:**
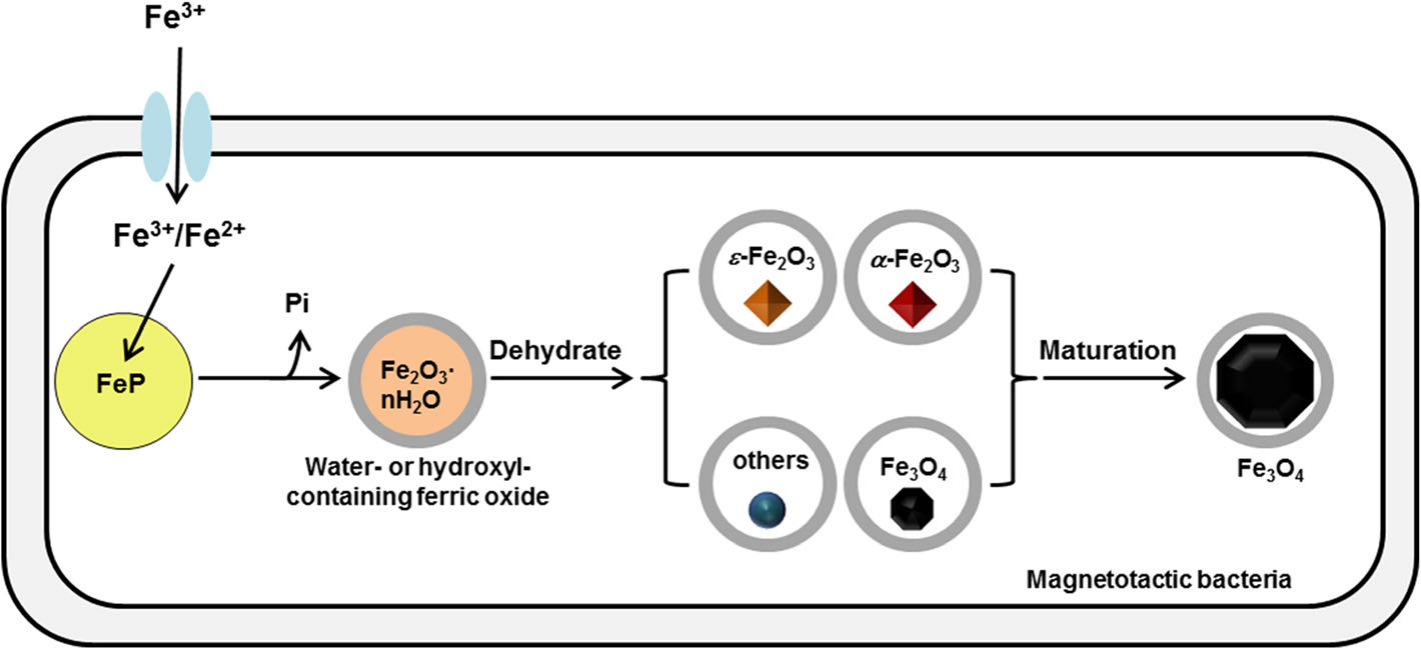
Schematic chemical route of magnetite biomineralization in MSR-1. Magnetosome biomineralization occurs through the following steps: Iron uptake (store as a phosphate-rich ferric hydroxide phase), dephosphorization, transformation of iron ion into magnetosome vesicle, formation of magnetosome intermediates that composed of various kinds of iron oxides and formation of mature magnetosomes composed of magnetite. Gray circles in the figure refer to magnetosome membrane

The chemical synthesis of highly crystalline magnetite requires harsh conditions, and the ability of organisms to form such particles rapidly under moderate conditions remains an interesting question. Based on our current data and evidence from previous studies, a new chemical route model of magnetite biomineralization in MTB is proposed (Fig. 6): ferric ion is taken up into the cell and stored as a phosphate-rich ferric hydroxide phase [23]. After dephosphorization, ferric ion is transferred to a magnetosome vesicle to form water- or hydroxyl-containing ferric oxide, followed by transformation into a variety of iron oxides that act as the precursors of mature magnetosomes, including Fe_3_O_4_, *α*-Fe_2_O_3_, *ε*-Fe_2_O_3_ and other phases that have not yet been defined. The mature magnetite crystals finally form from these intermediate phases.

## Additional file

Additional file 1: **Figure S1.** HRTEM images with high resolution and the corresponding FFT analyses of intracellular iron oxide nanoparticles in MSR-1 wild type after the induction of ferric citrate for different time interval. **Figure S2.** Some representative HRTEM images of epsilon-Fe_2_O_3_ and alpha-Fe_2_O_3_ used for the phase ratio determination in Fig. 5 of the main text. **Table S1.** Crystallographic information of the *ε*-Fe_2_O_3_ exhibited in Fig. 3a, Fig. 4 and the theoretical data of *ε*-Fe_2_O_3_ and Fe_3_O_4_ (magnetite). For further crystallographic information, people can refer to pdf card as 33–0664 for *α*-Fe_2_O_3_, 65–3107 for magnetite and 52–1449 for *ε*-Fe_2_O_3_, respectively. (DOCX 19846 kb)

## Data Availability

All data generated and analyzed in this study are available from the corresponding author on request.
