# Supplementary material for: Epsilon-Fe2O3 is a novel intermediate for magnetite biosynthesis in magnetotactic bacteria
Source: Biomater Res. 2019 Aug 2;23:13. doi: 10.1186/s40824-019-0162-1 (PMC6679552; doi:10.1186/s40824-019-0162-1)
Supplement: Supplementary file 1 — Figure S1. HRTEM images with high resolution and the corresponding FFT analyses of intracellular iron oxide nanoparticles in MSR-1 wild type after the induction of ferric citrate for different time interval. Figure S2. Some representative HRTEM images of epsilon-Fe2O3 and alpha-Fe2O3 used for the phase ratio determination in Fig. 5 of the main text. Table S1. Crystallographic information of the ε-Fe2O3 exhibited in Fig. 3a, Fig. 4 and the theoretical data of ε-Fe2O3 and Fe3O4 (magnetite). For further crystallographic information, people can refer to pdf card as 33–0664 for α-Fe2O3, 65–3107 for magnetite and 52–1449 for ε-Fe2O3, respectively. (DOCX 19846 kb) [file 40824_2019_162_MOESM1_ESM.docx]

**Supplementary Information:**

**Epsilon-Fe_2_O_3_ is a novel intermediate for magnetite biosynthesis in magnetotactic bacteria**

Tong Wen^1, 3, †^, Yunpeng Zhang^1, 4†^, Yuanyuan Geng^1^, Junquan Liu^1^, Abdul Basit^1^, Jiesheng Tian^1^, Ying Li^1^, Jilun Li^1^, Jing Ju^2*^, Wei Jiang^1*^

**Supplementary Information Figure S1.** HRTEM images with high resolution and the corresponding FFT analyses of intracellular iron oxide nanoparticles in MSR-1 wild type after the induction of ferric citrate for different time interval.


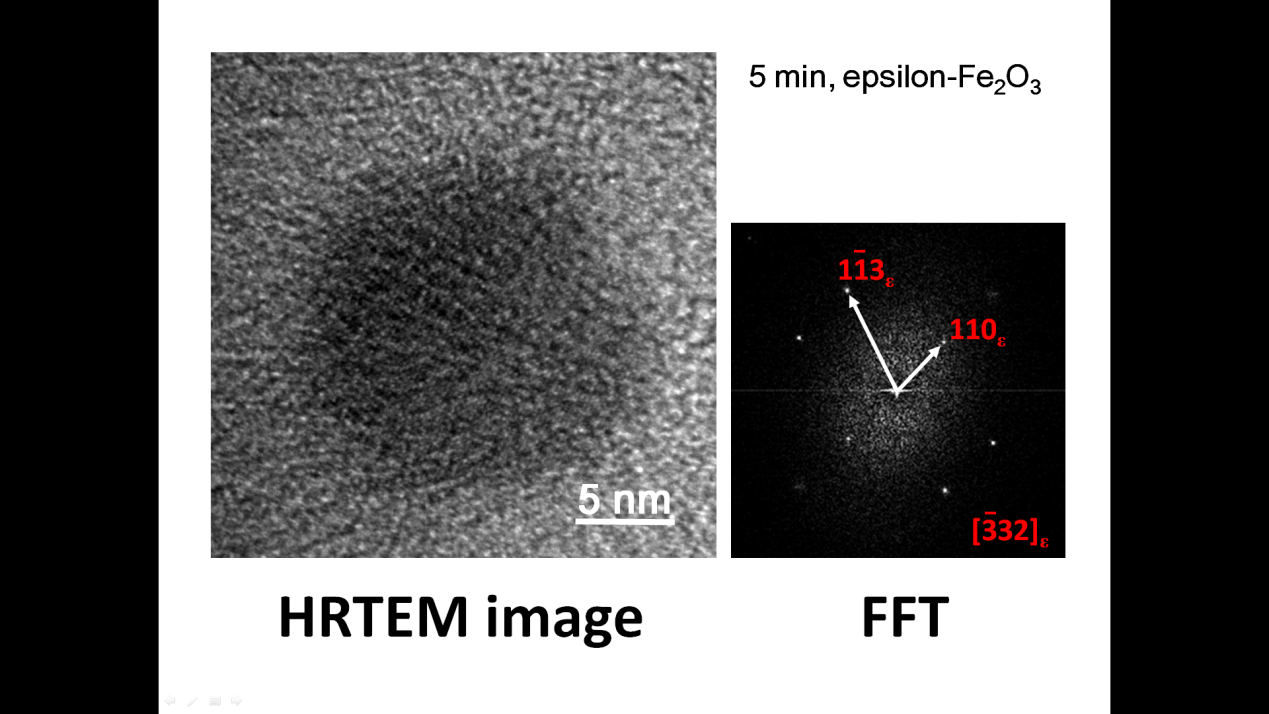


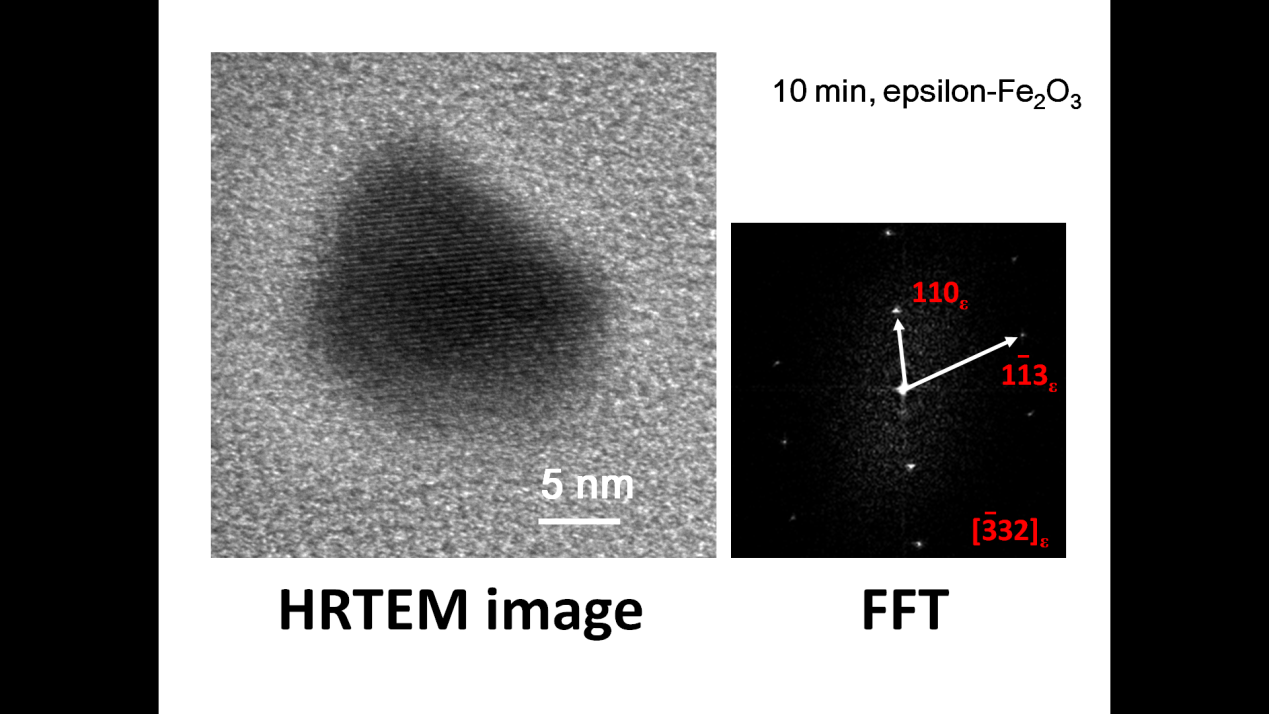


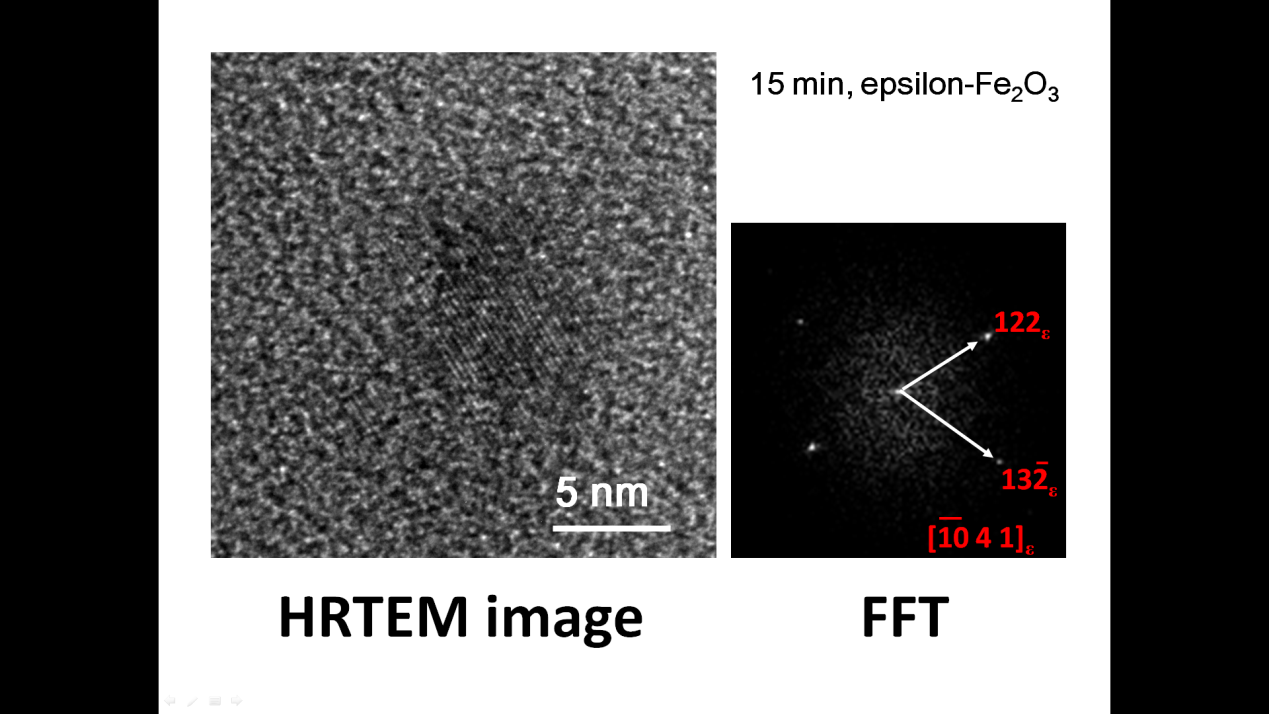


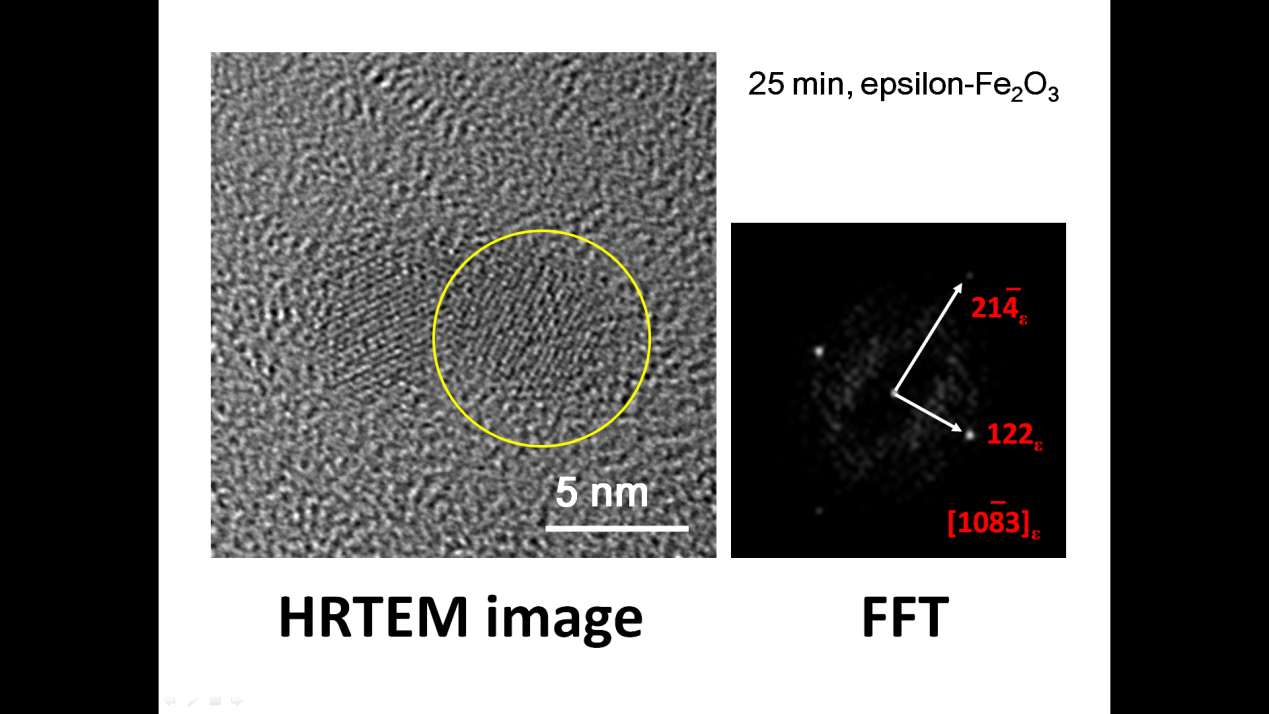


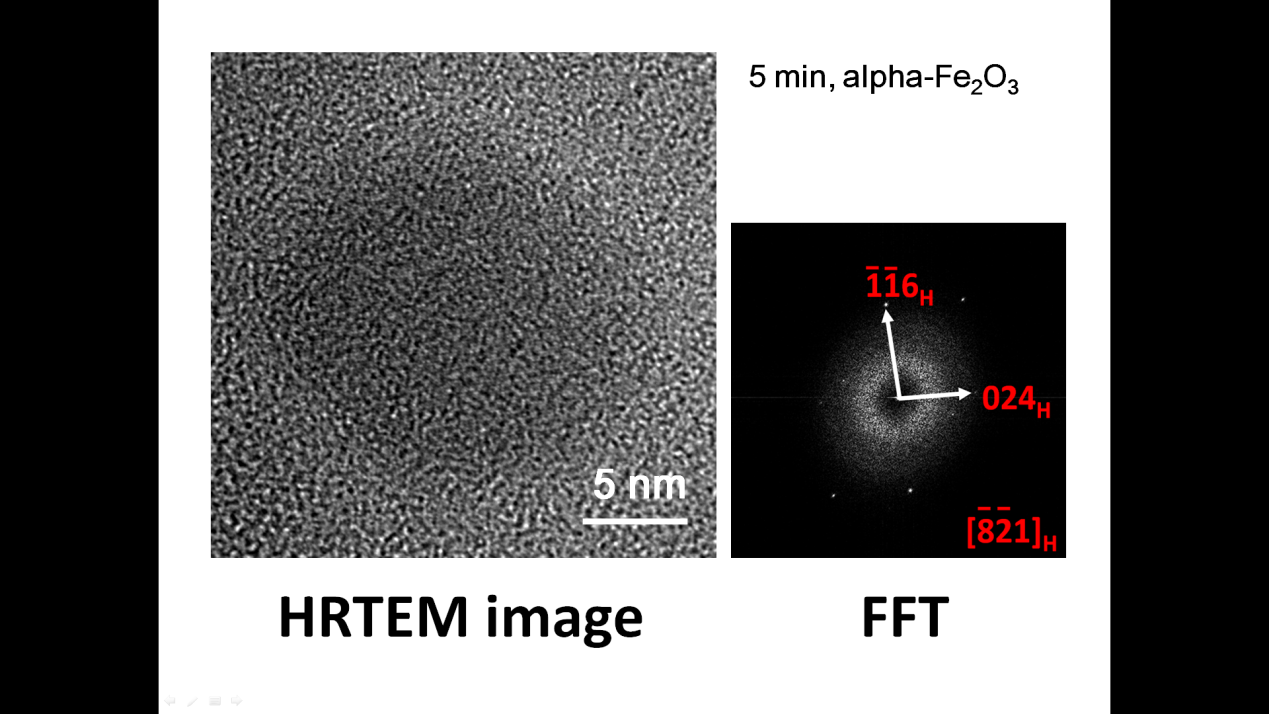


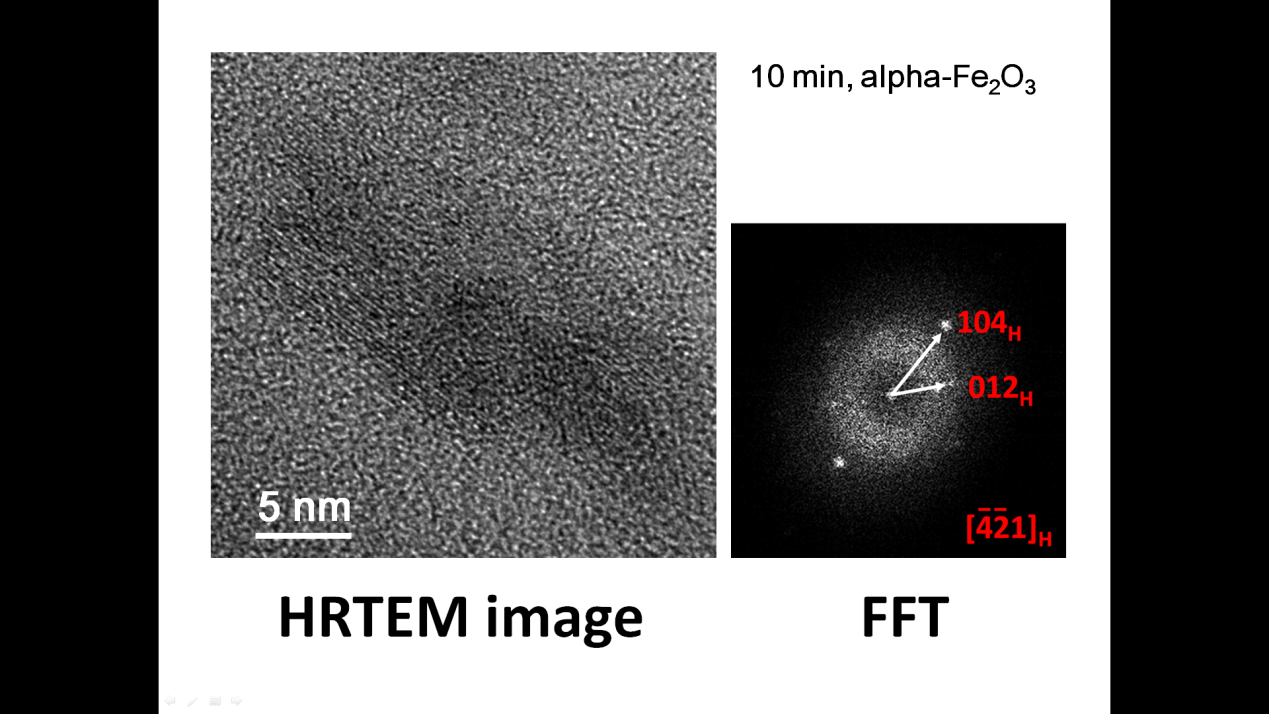


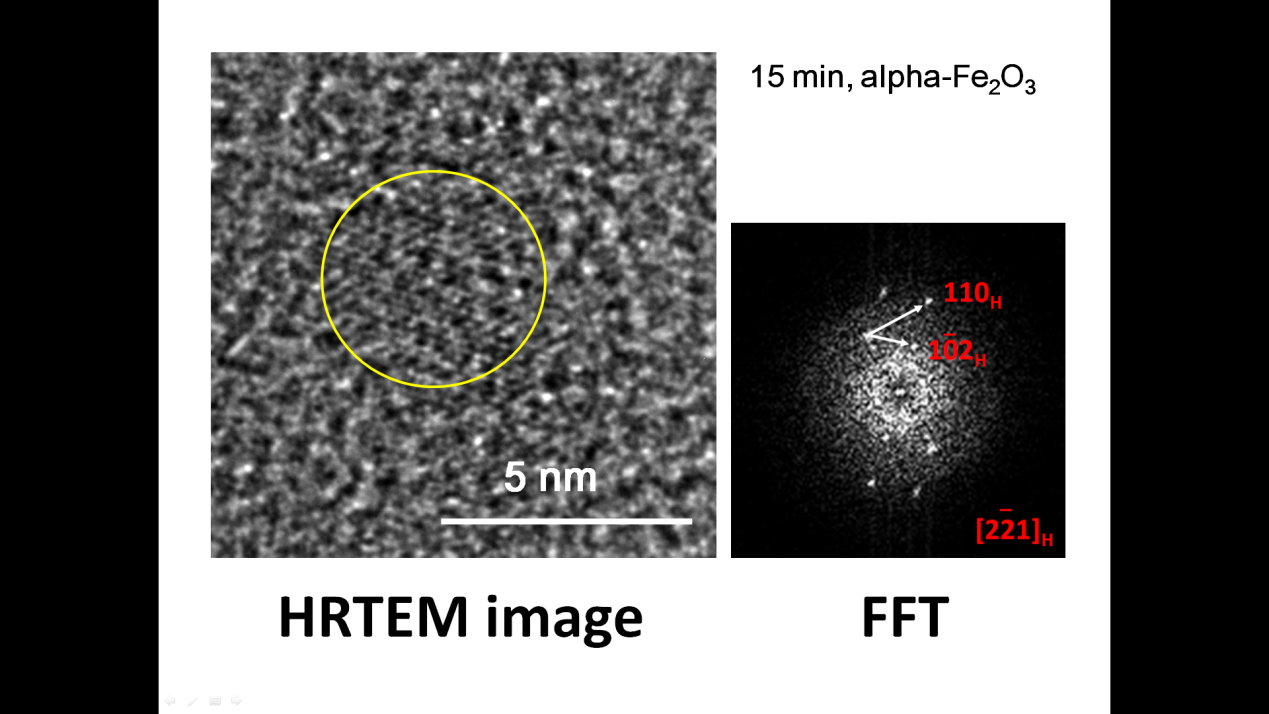


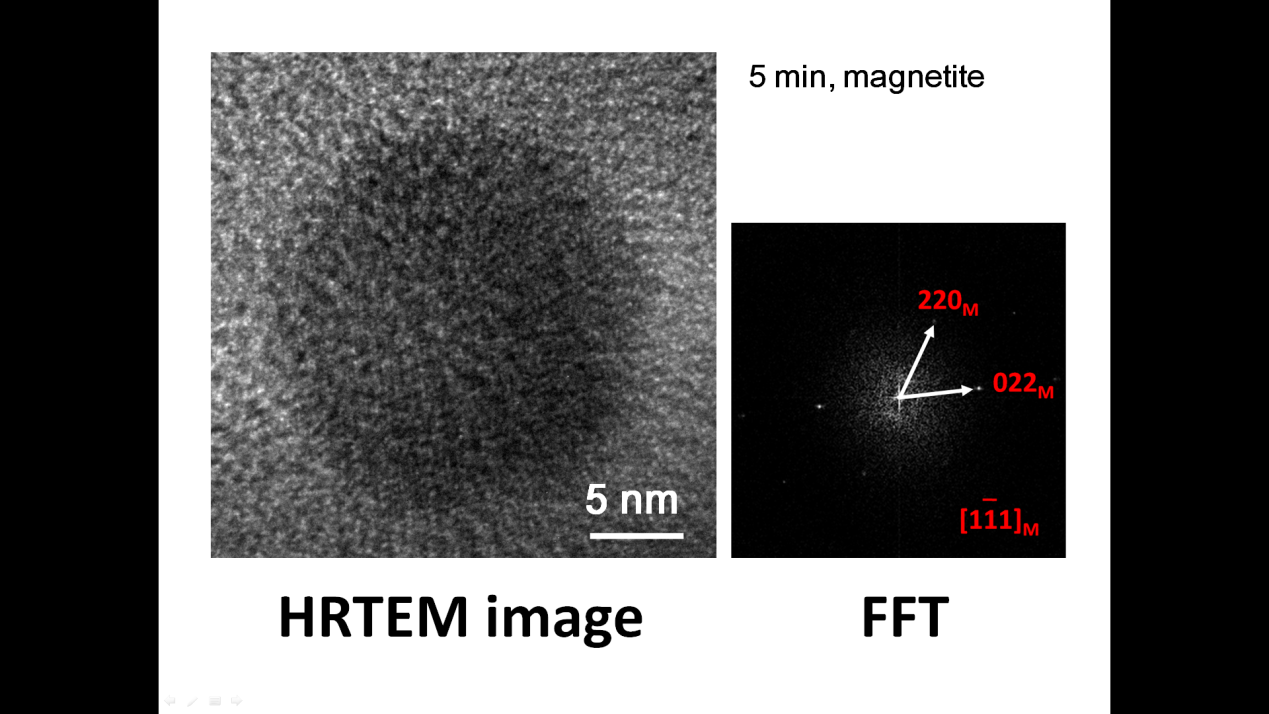


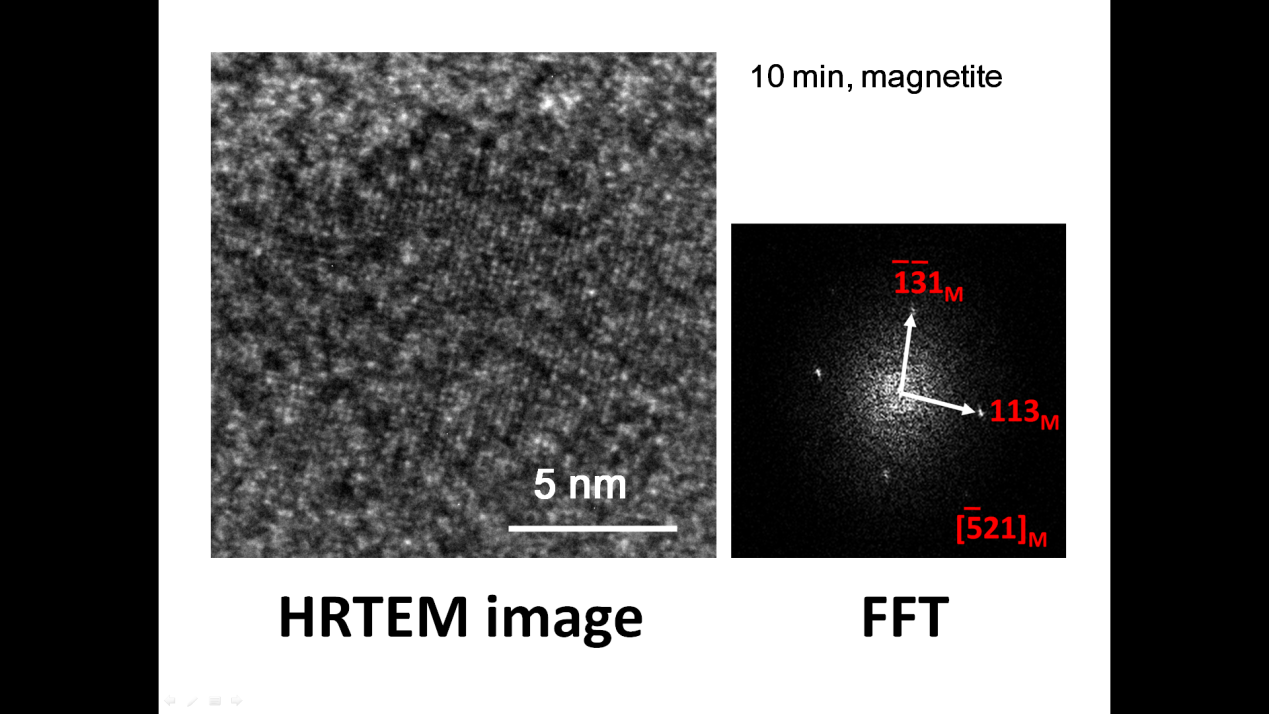


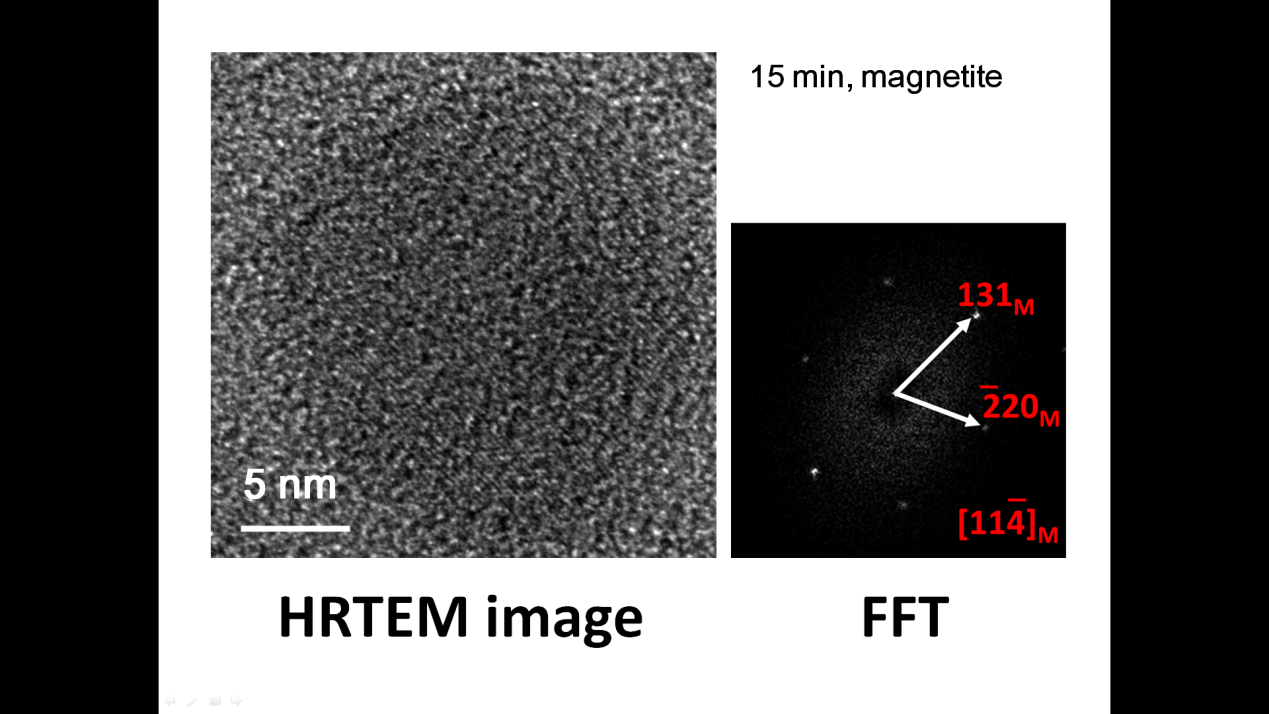


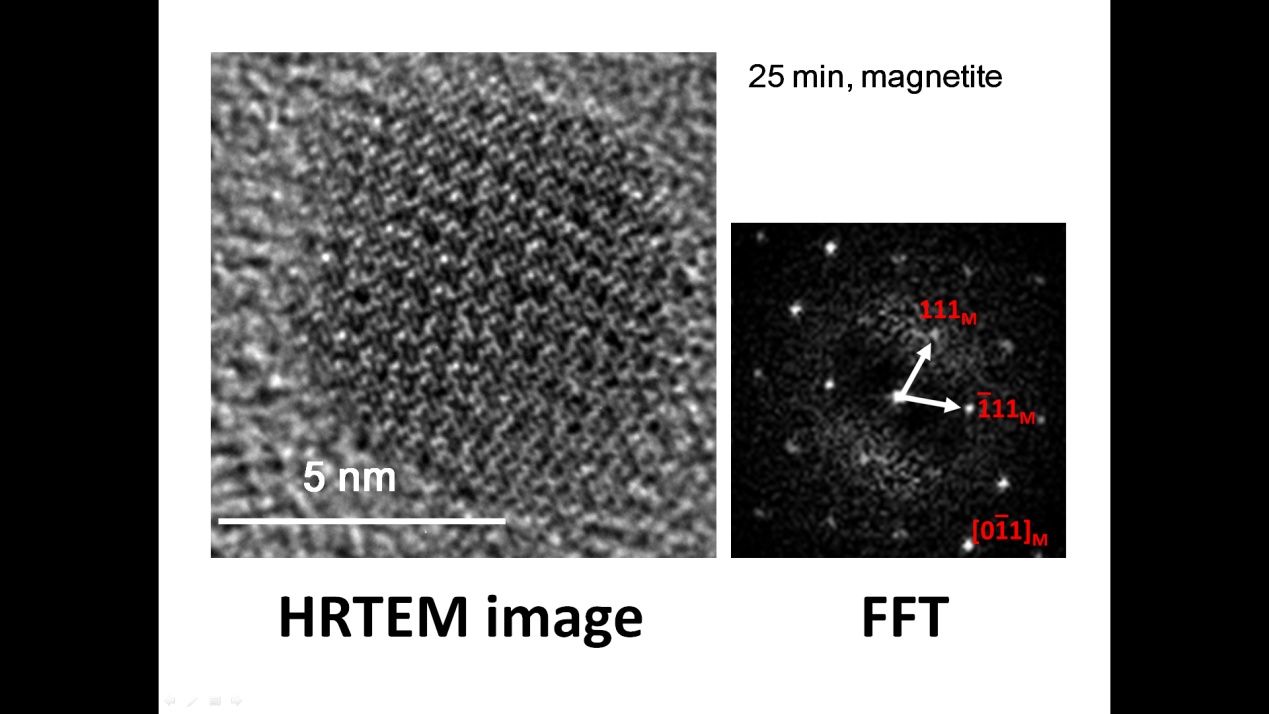


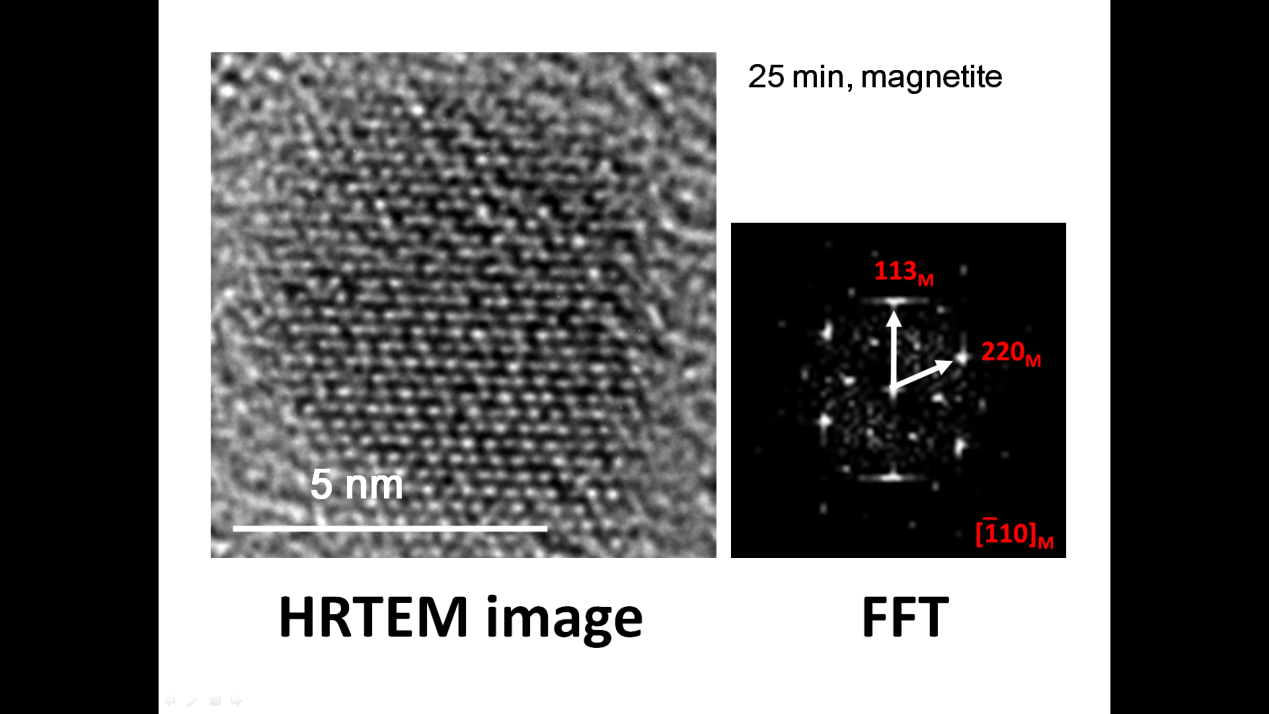


**Supplementary Information Figure S2**. Some representative HRTEM images of epsilon-Fe_2_O_3_ and alpha-Fe_2_O_3_ used for the phase ratio determination in Fig. 5 of the main text.


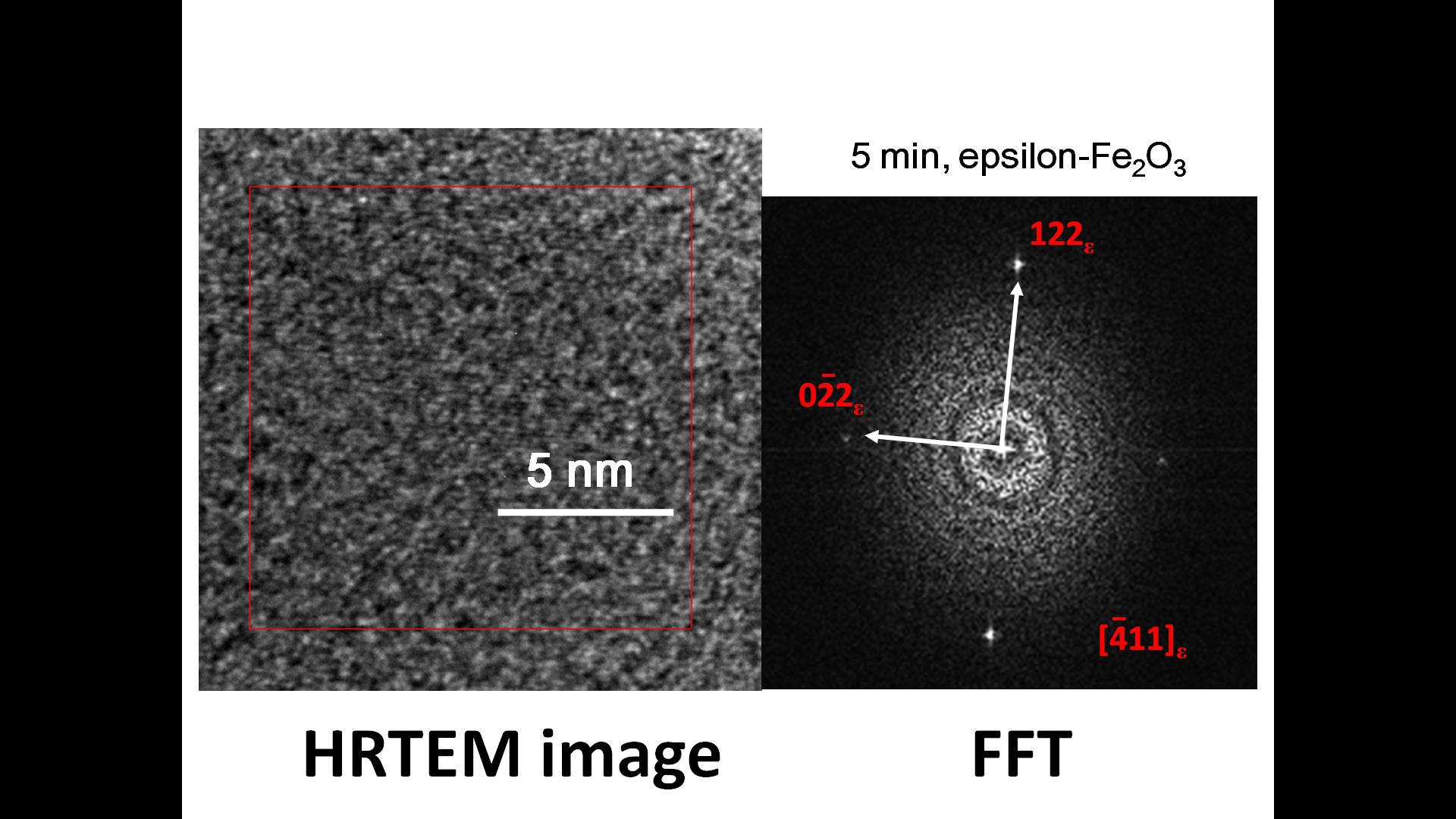

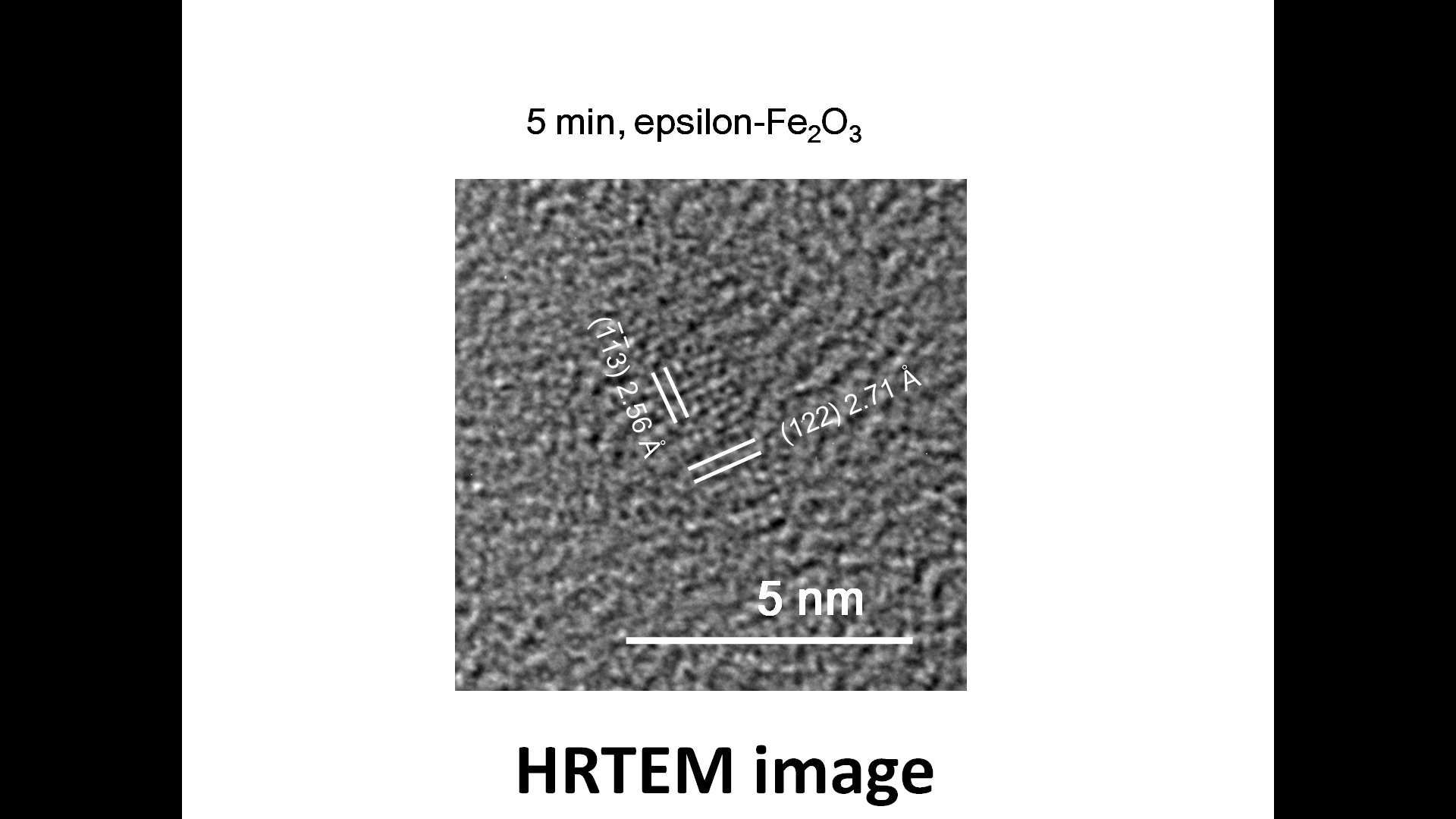

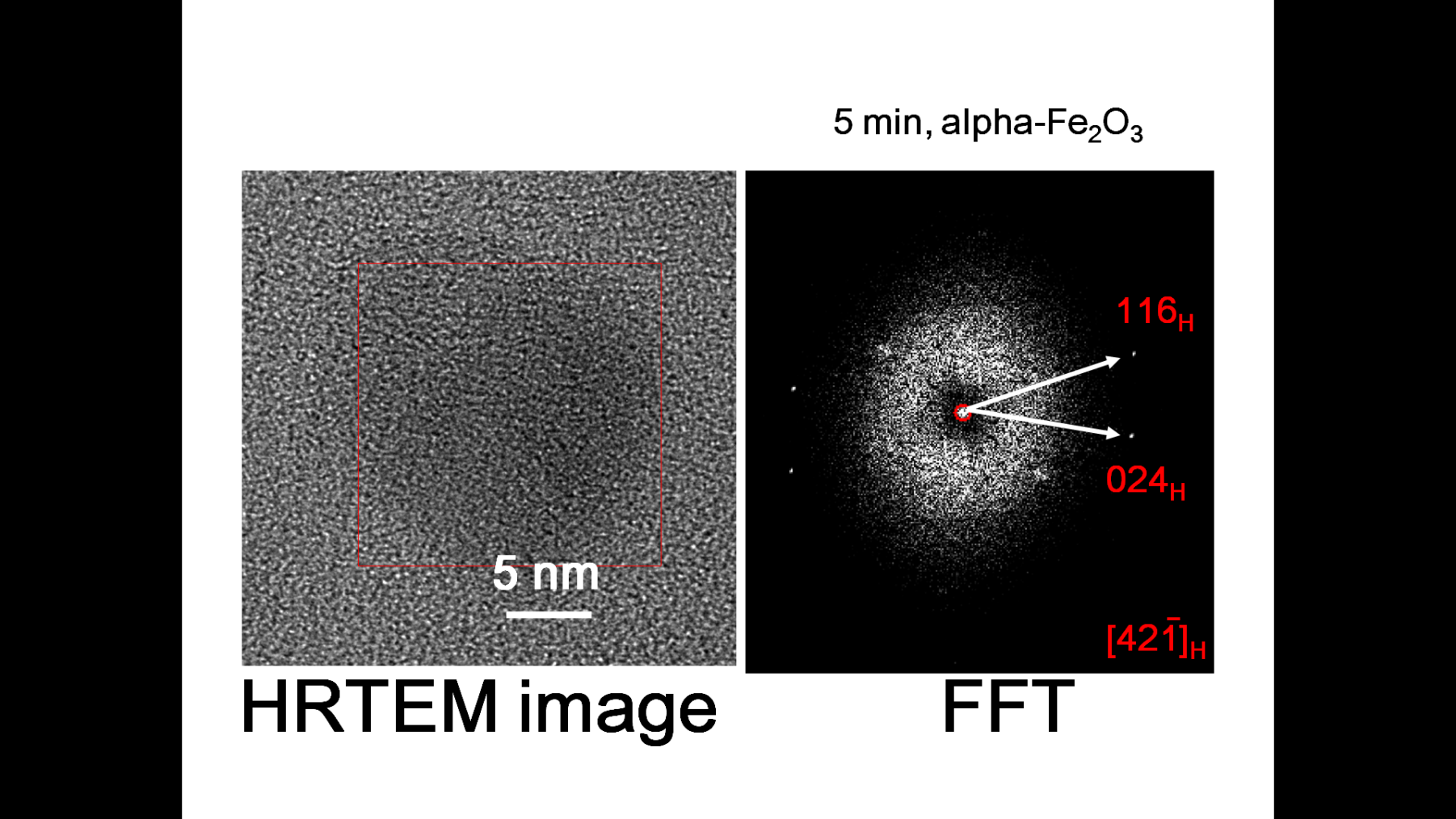

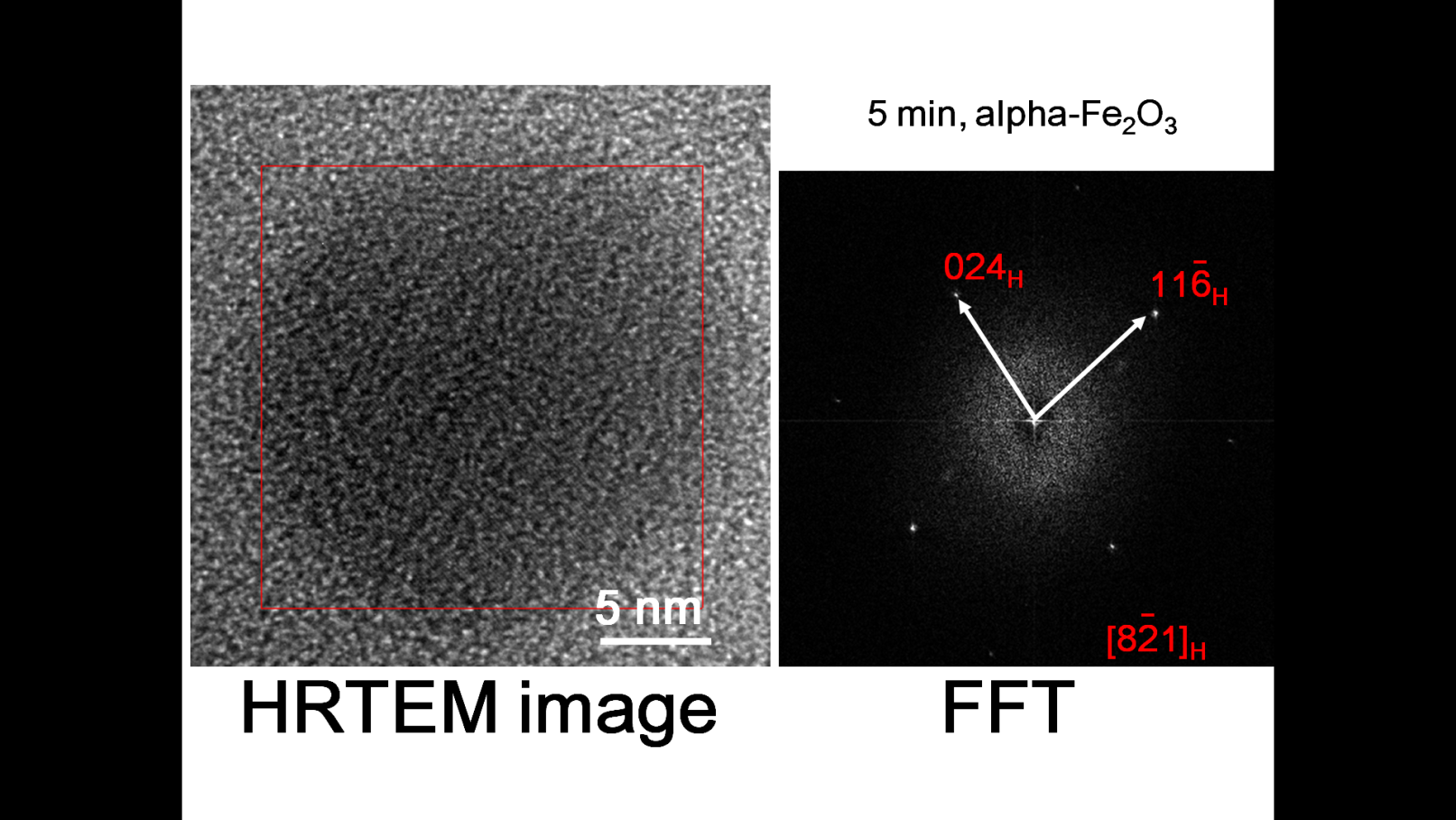

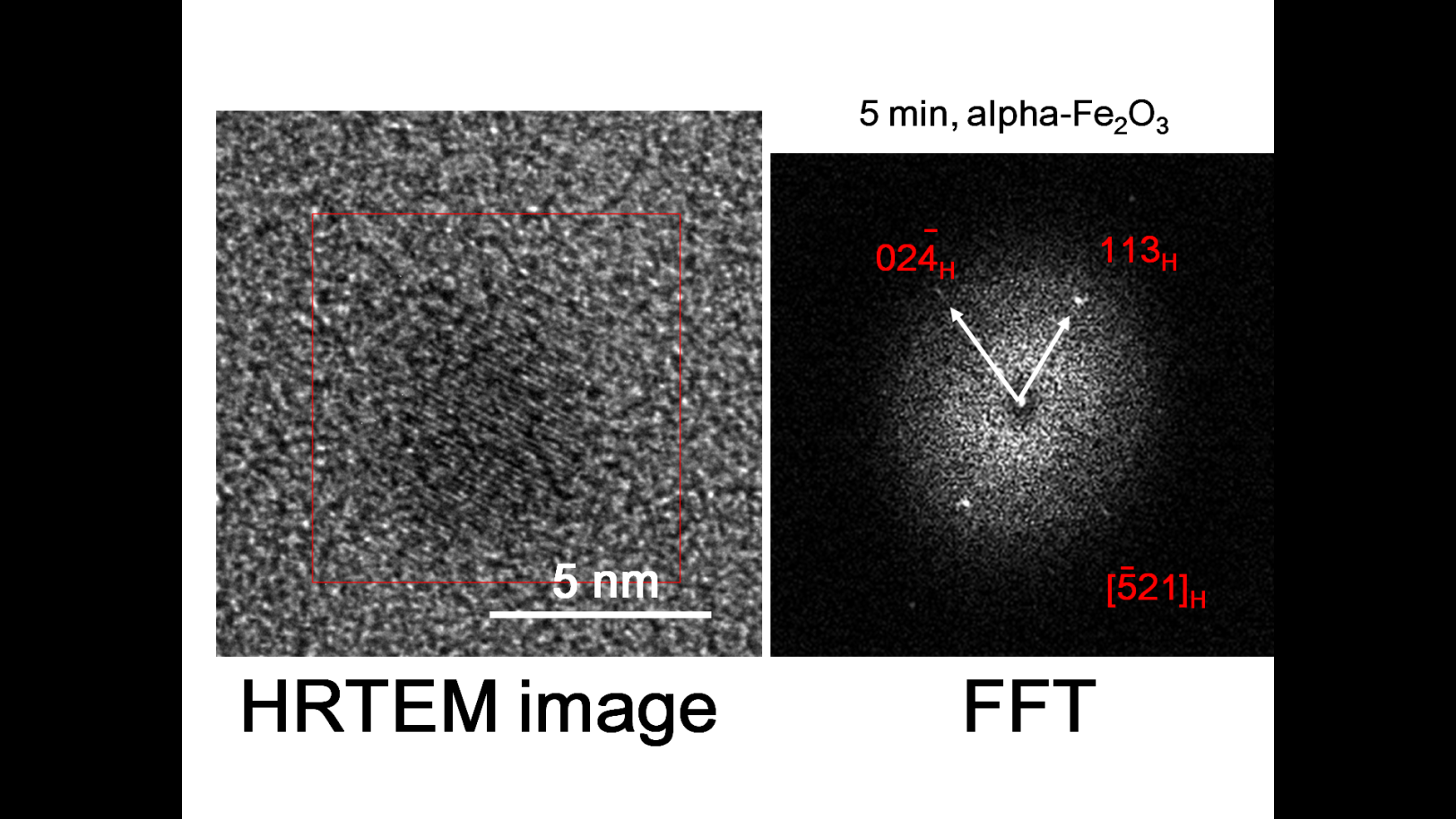

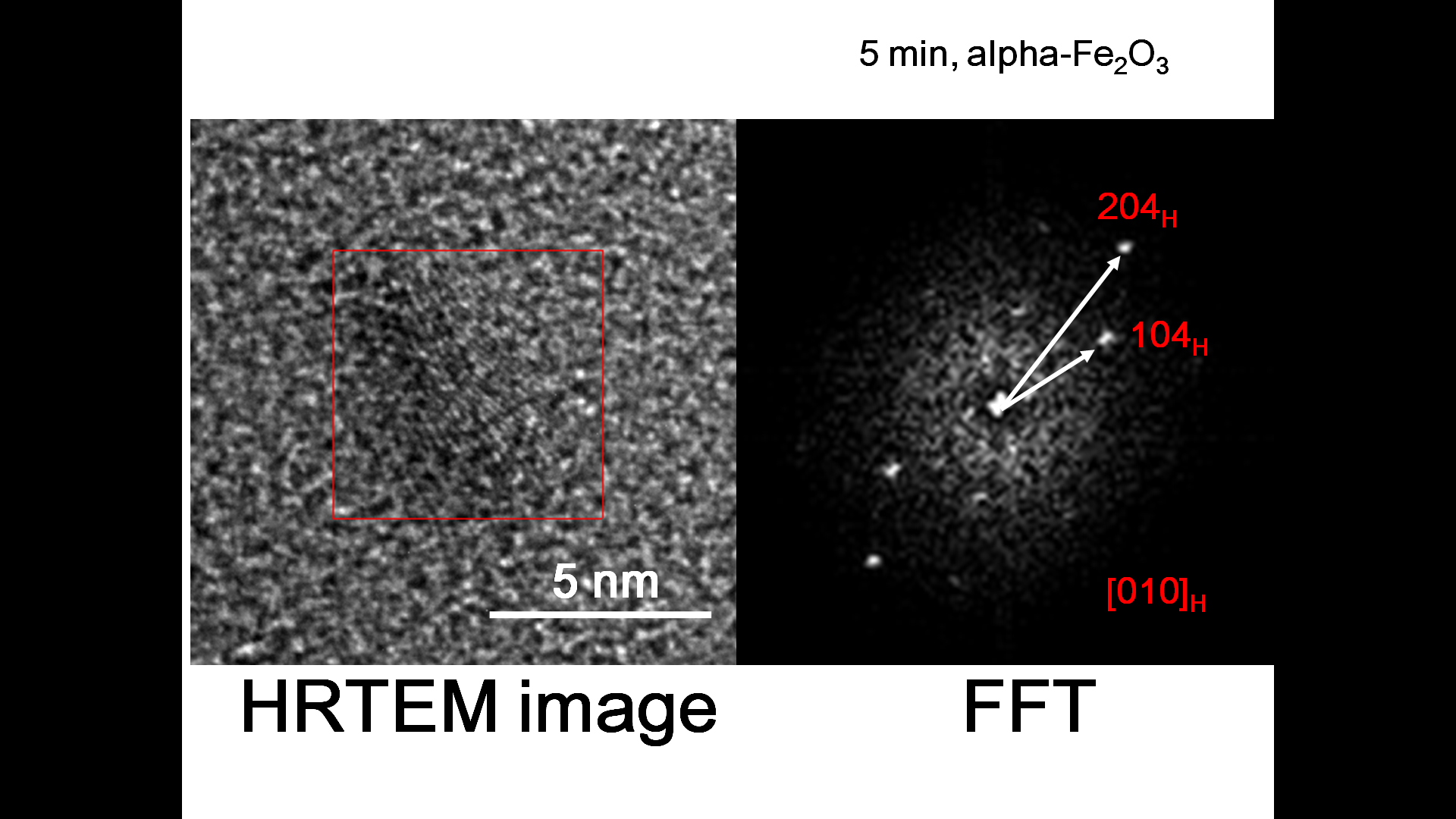

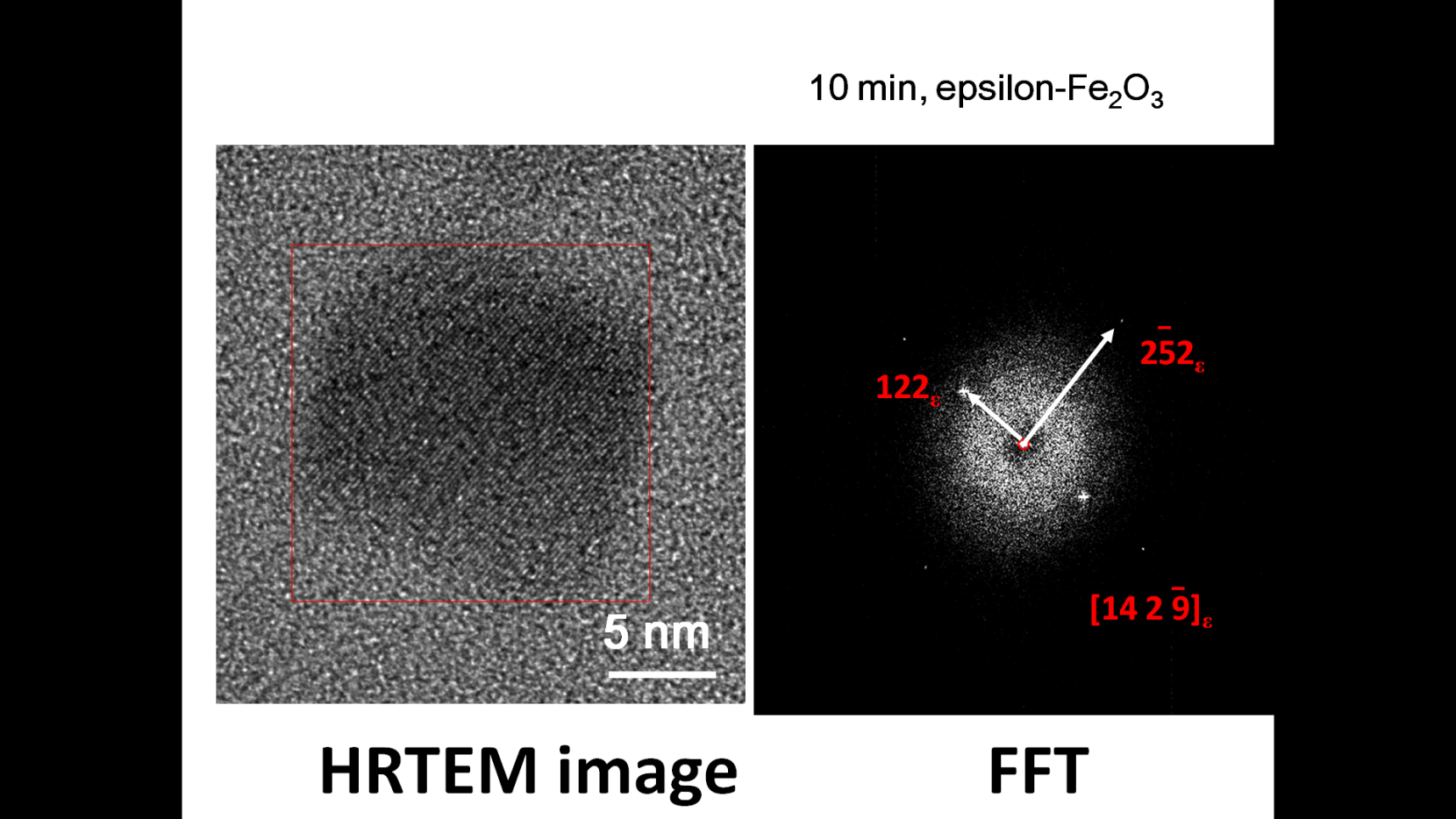

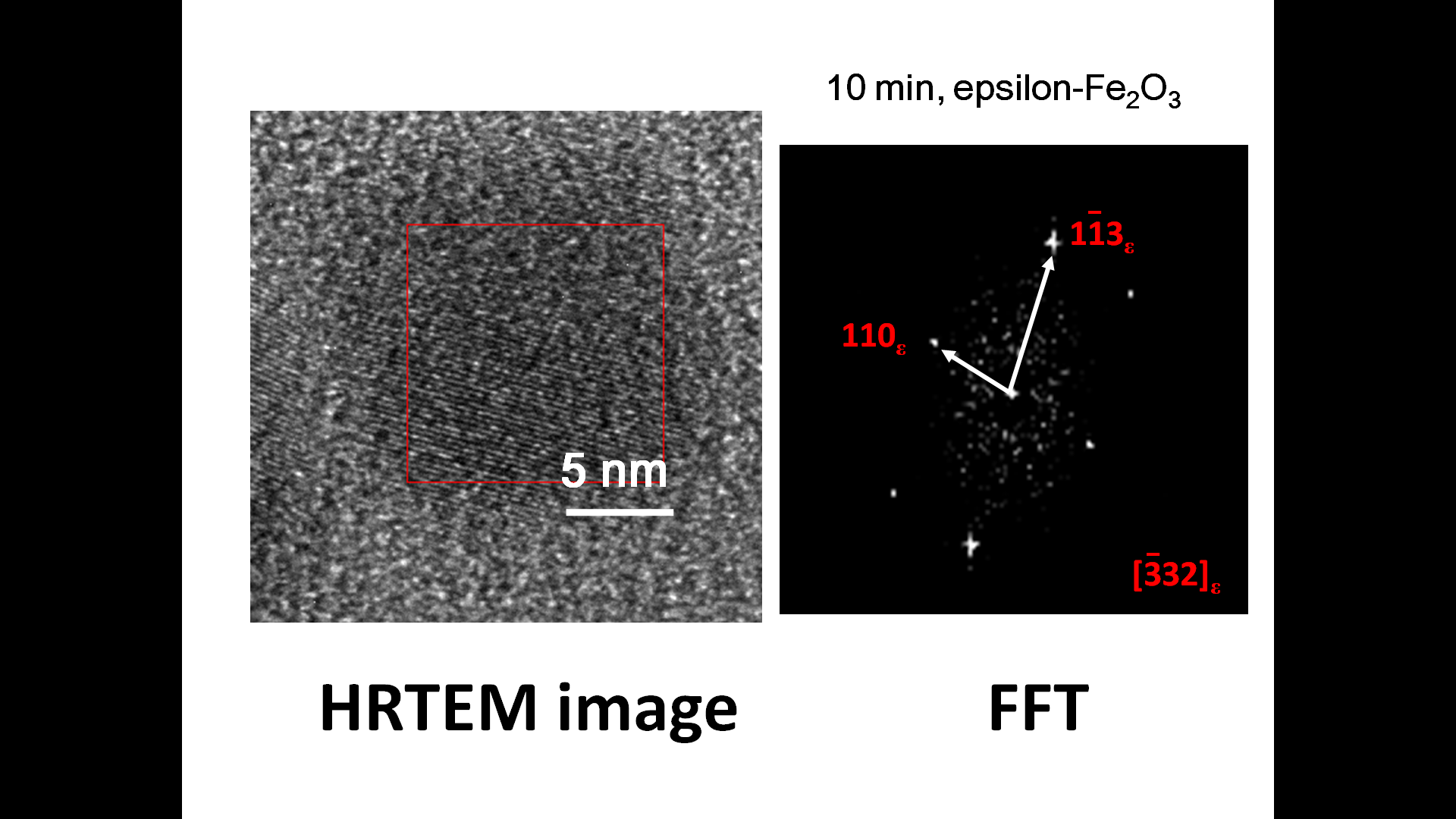

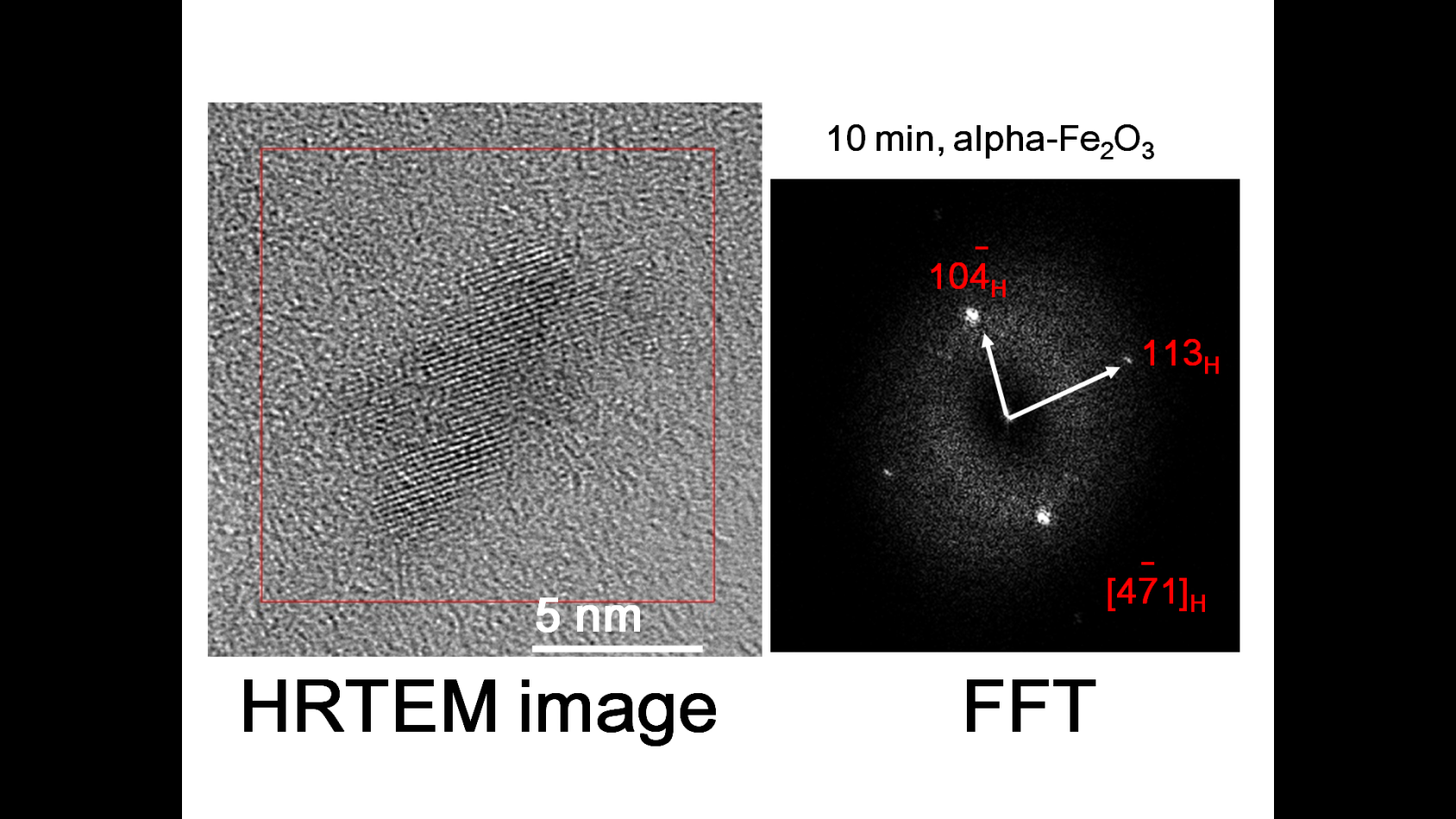

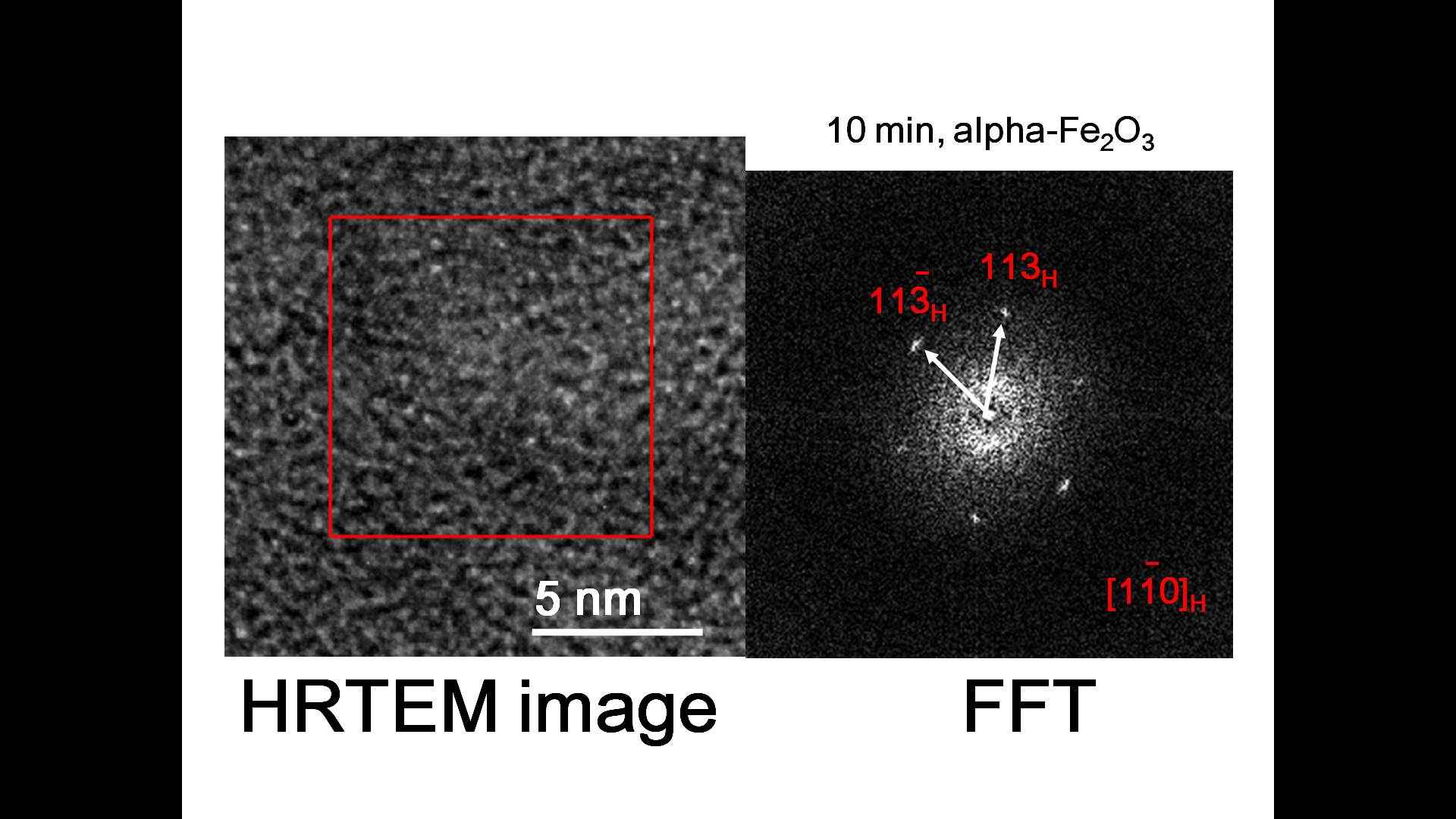

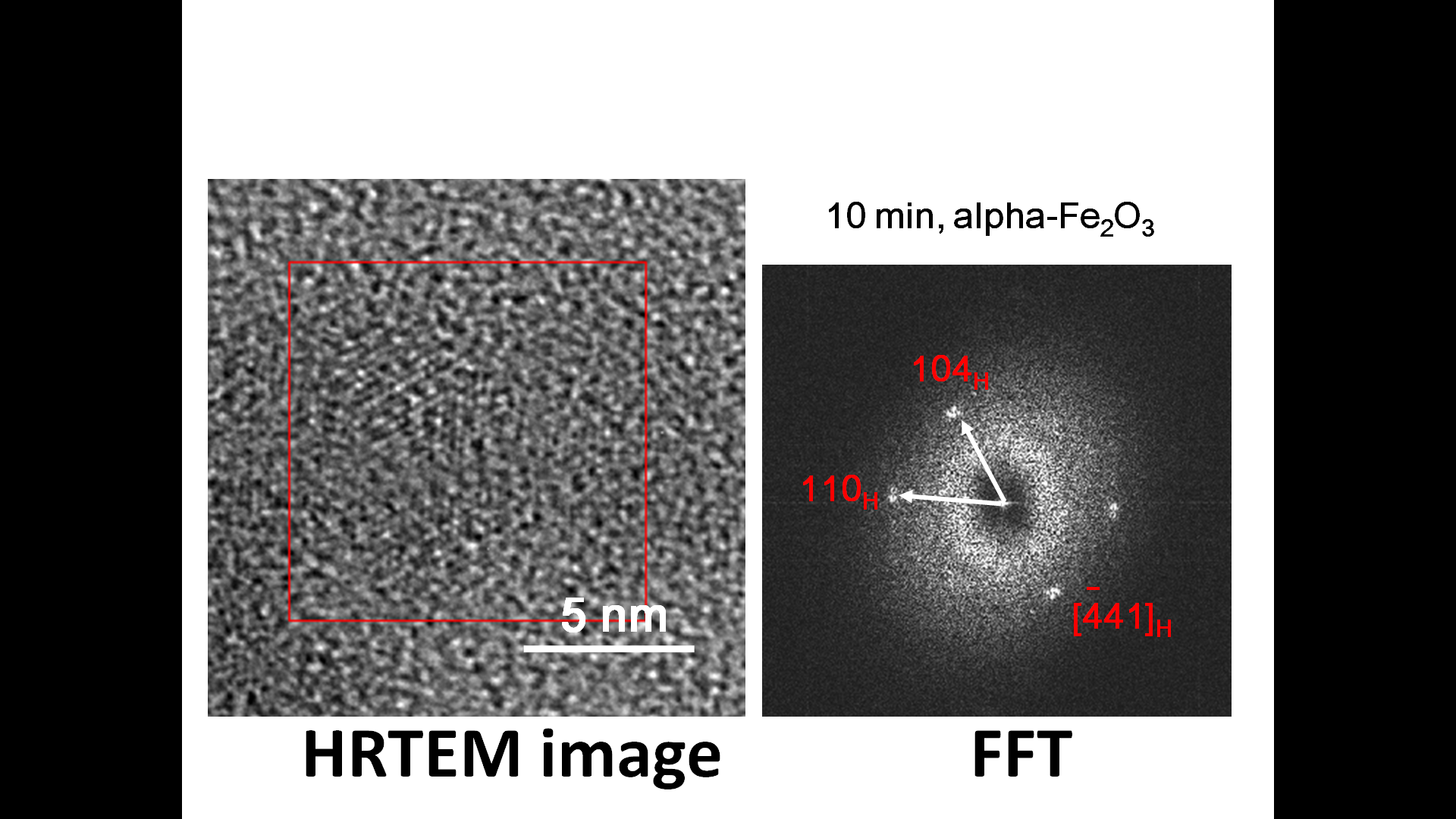

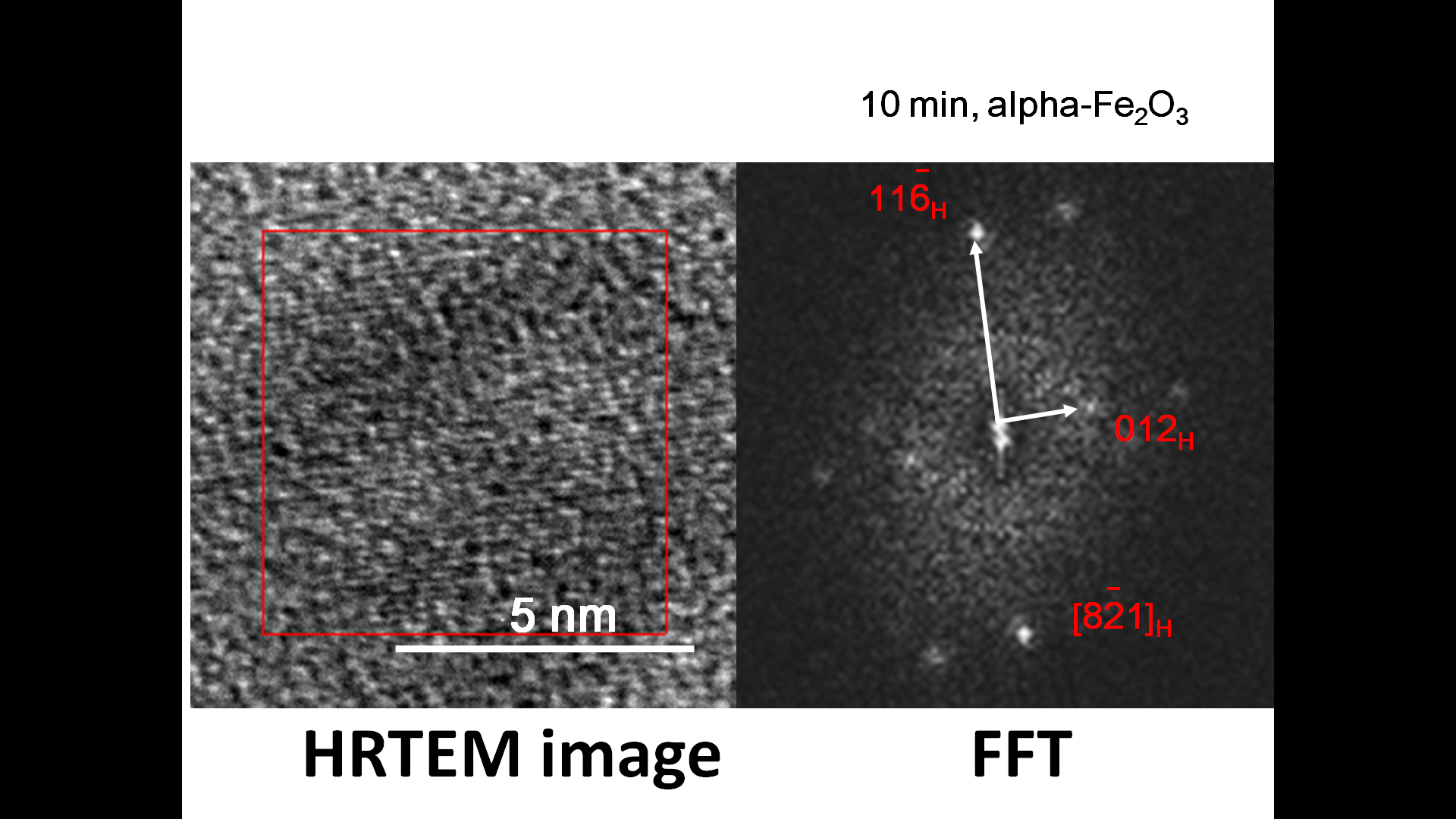

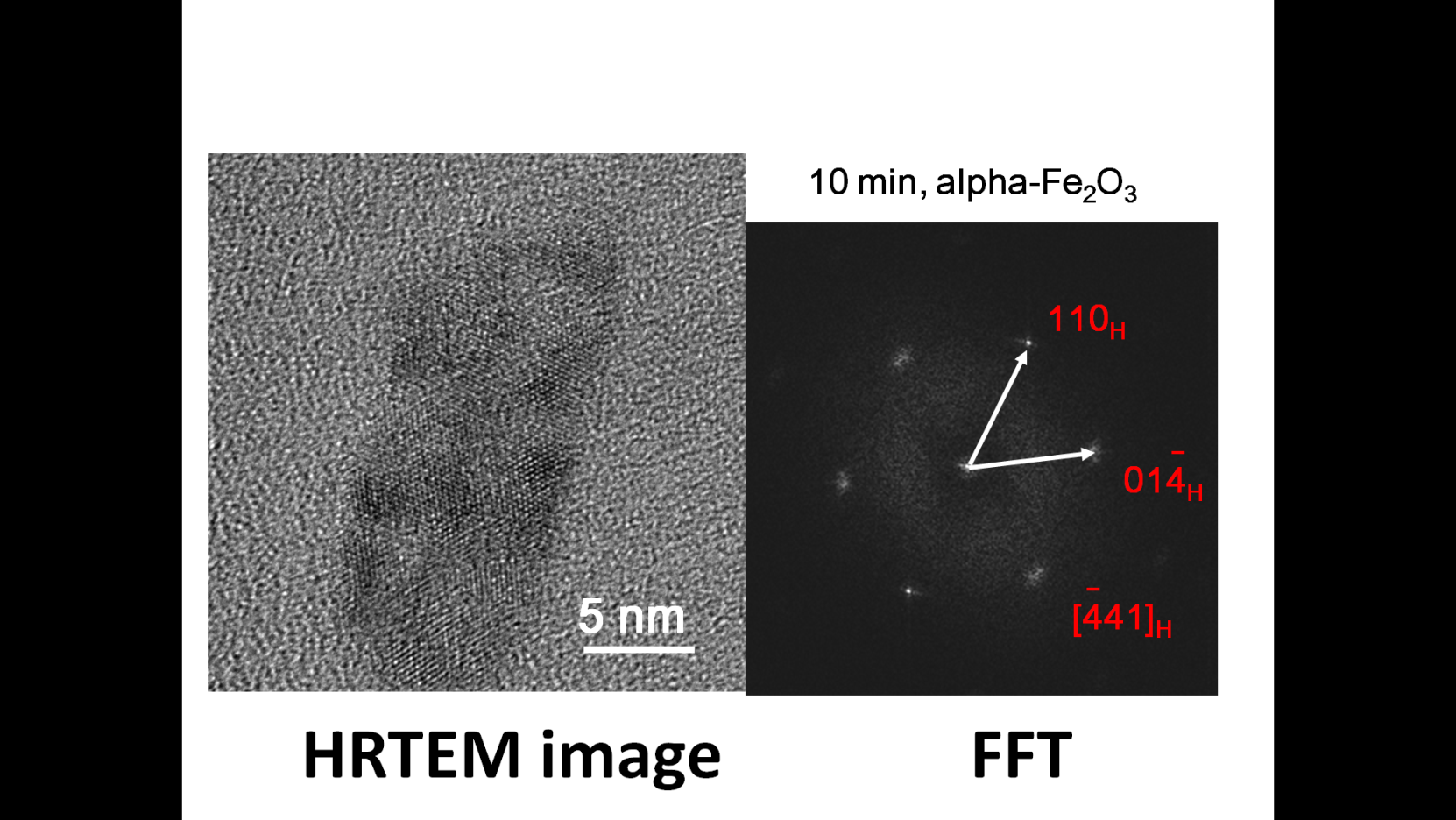

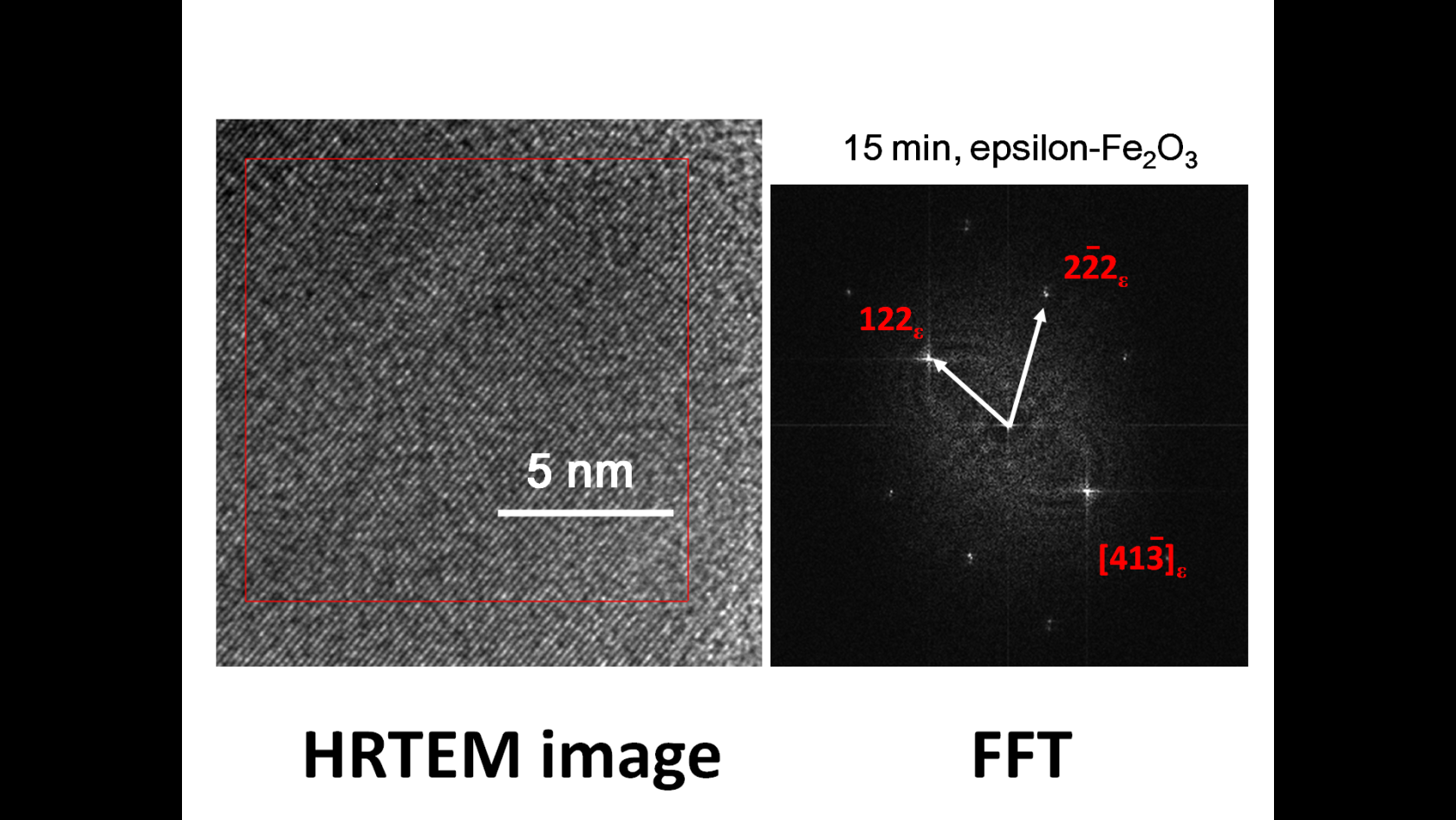

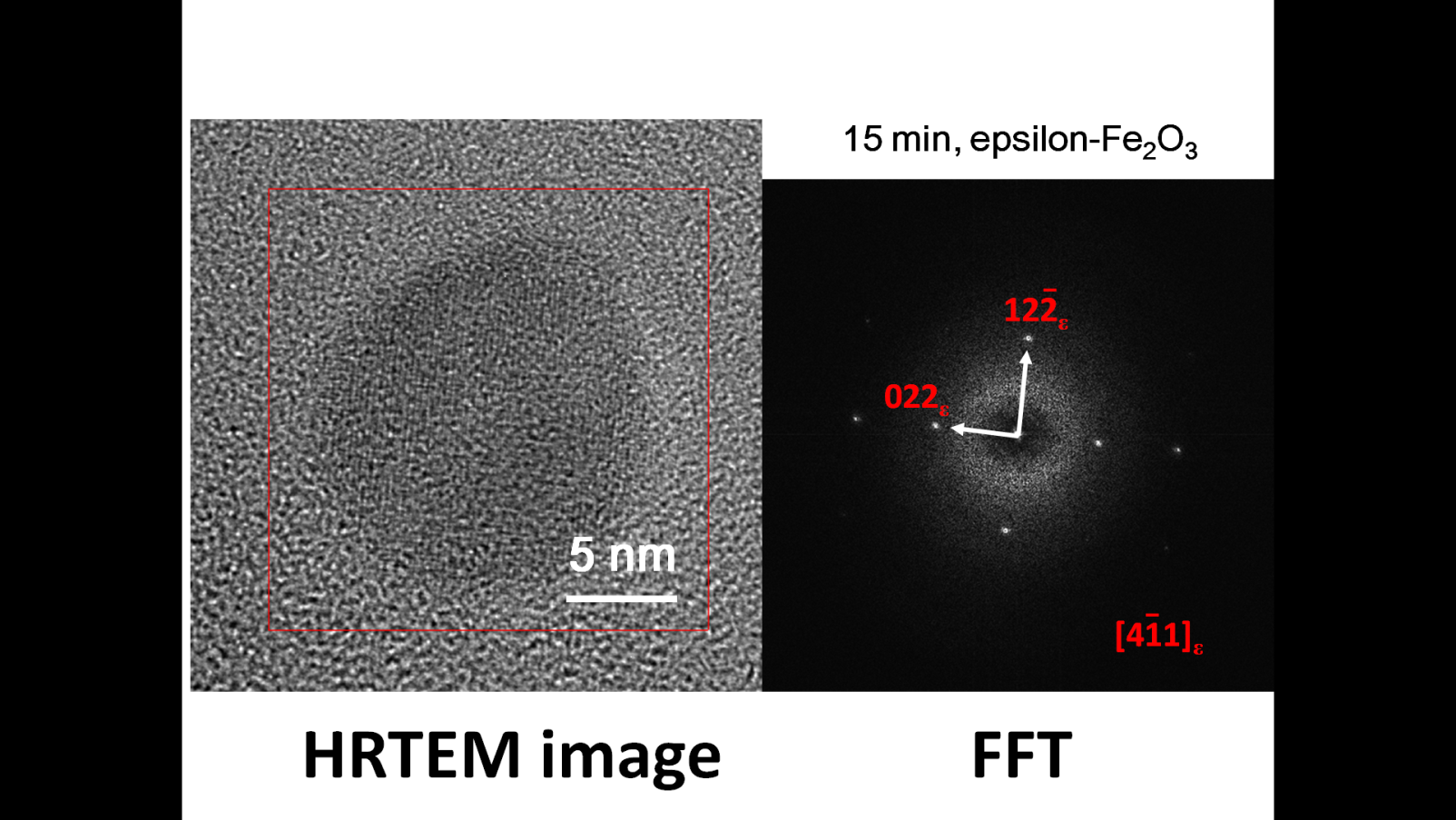

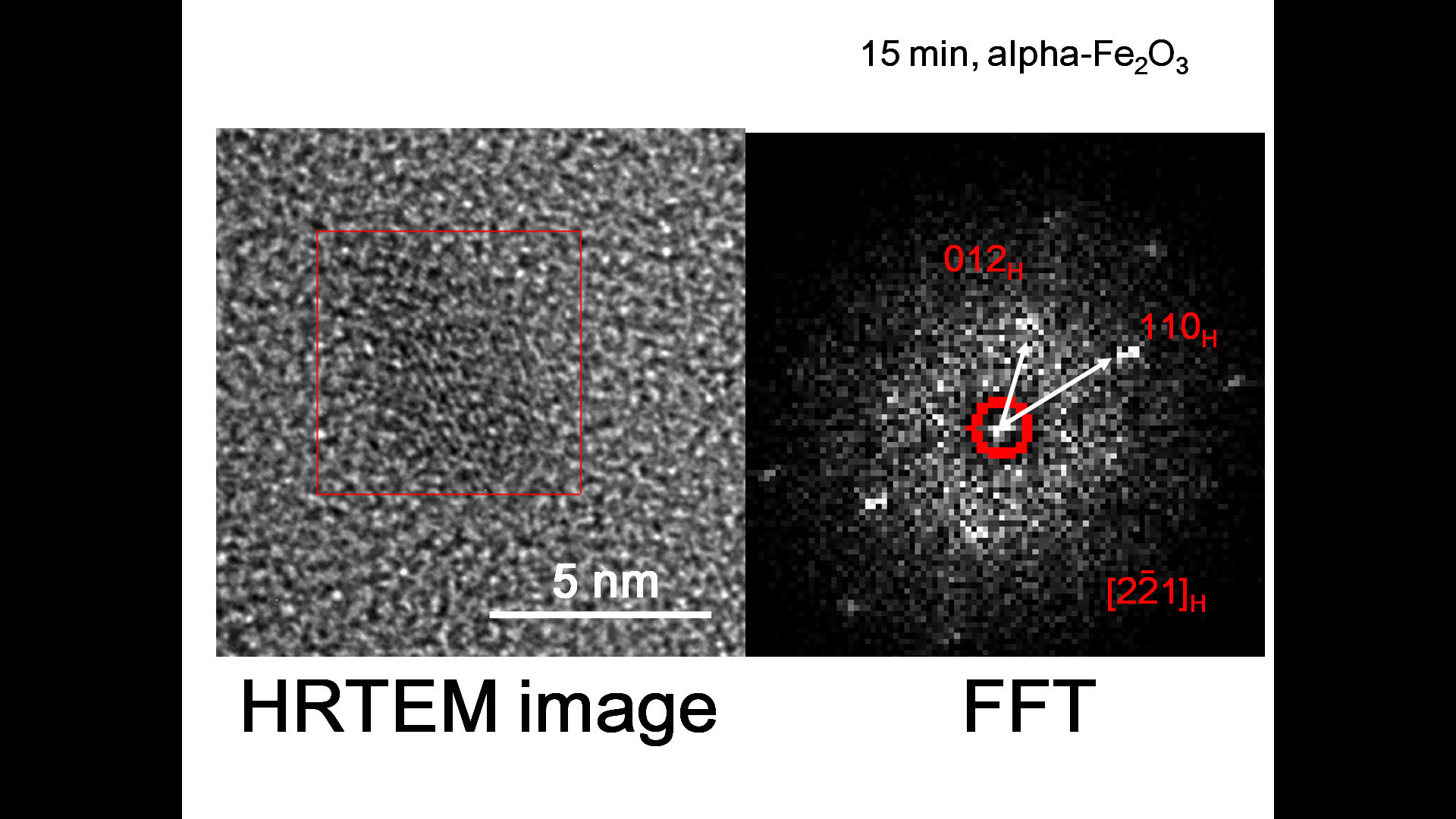

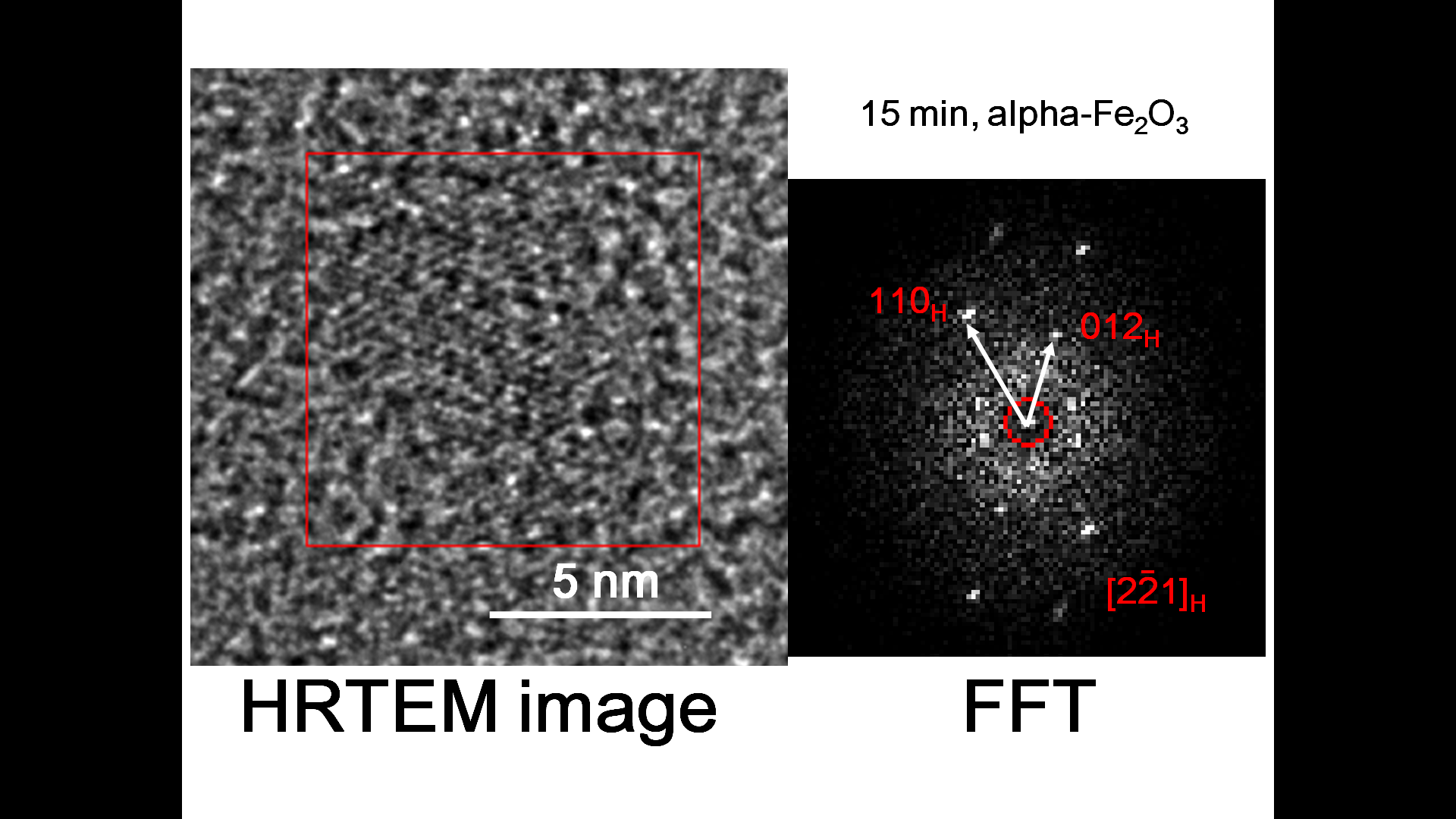

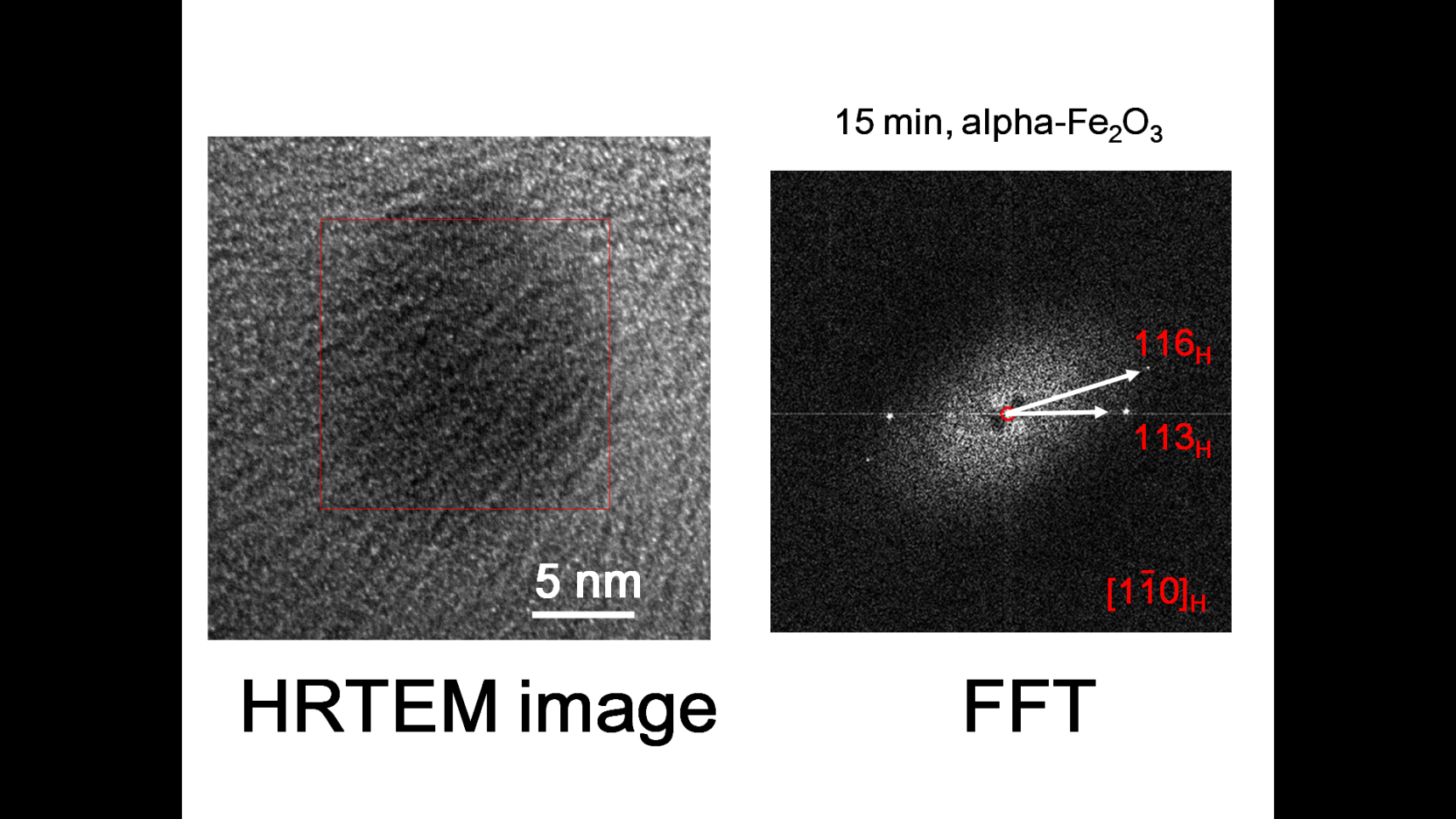

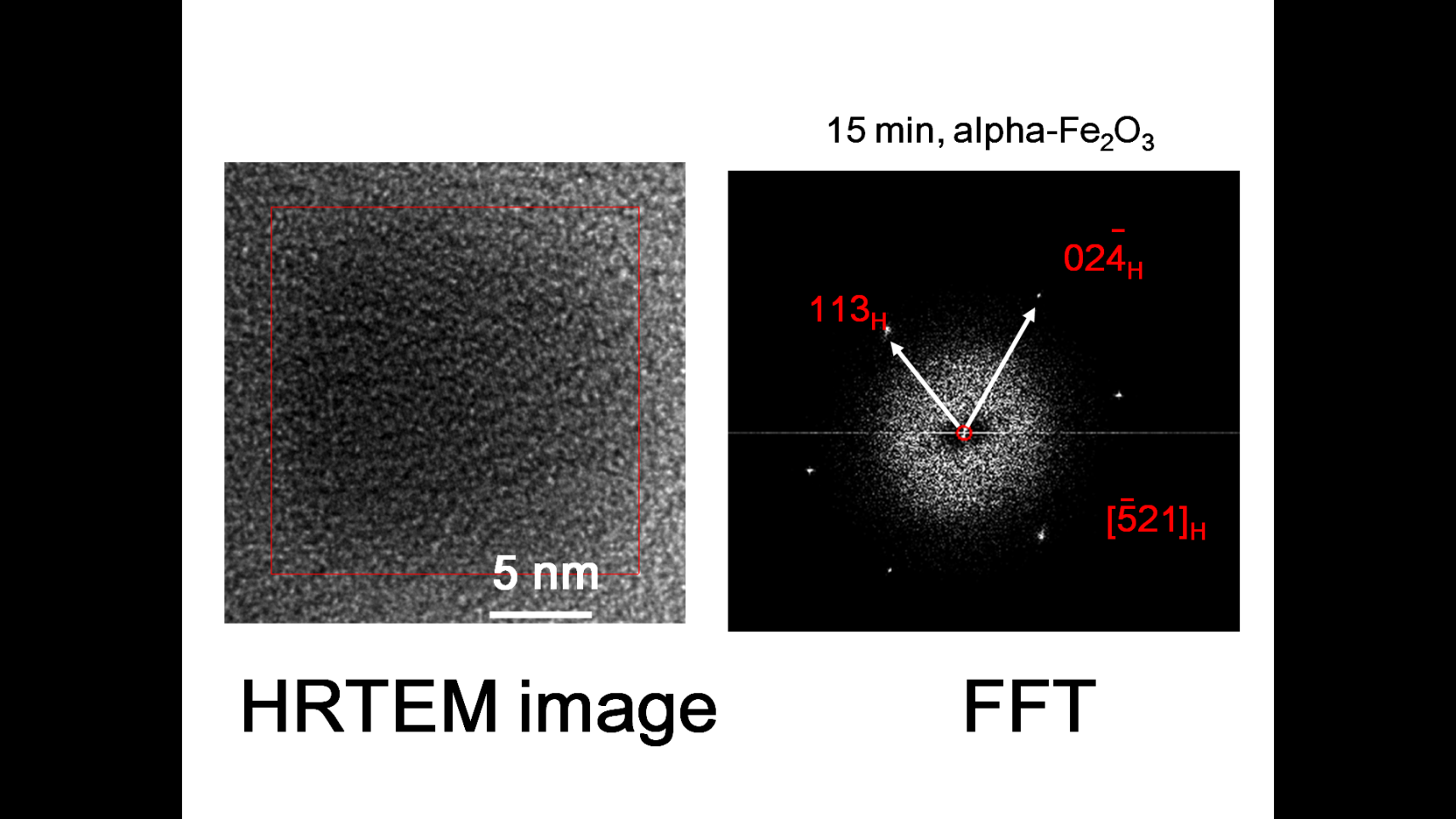

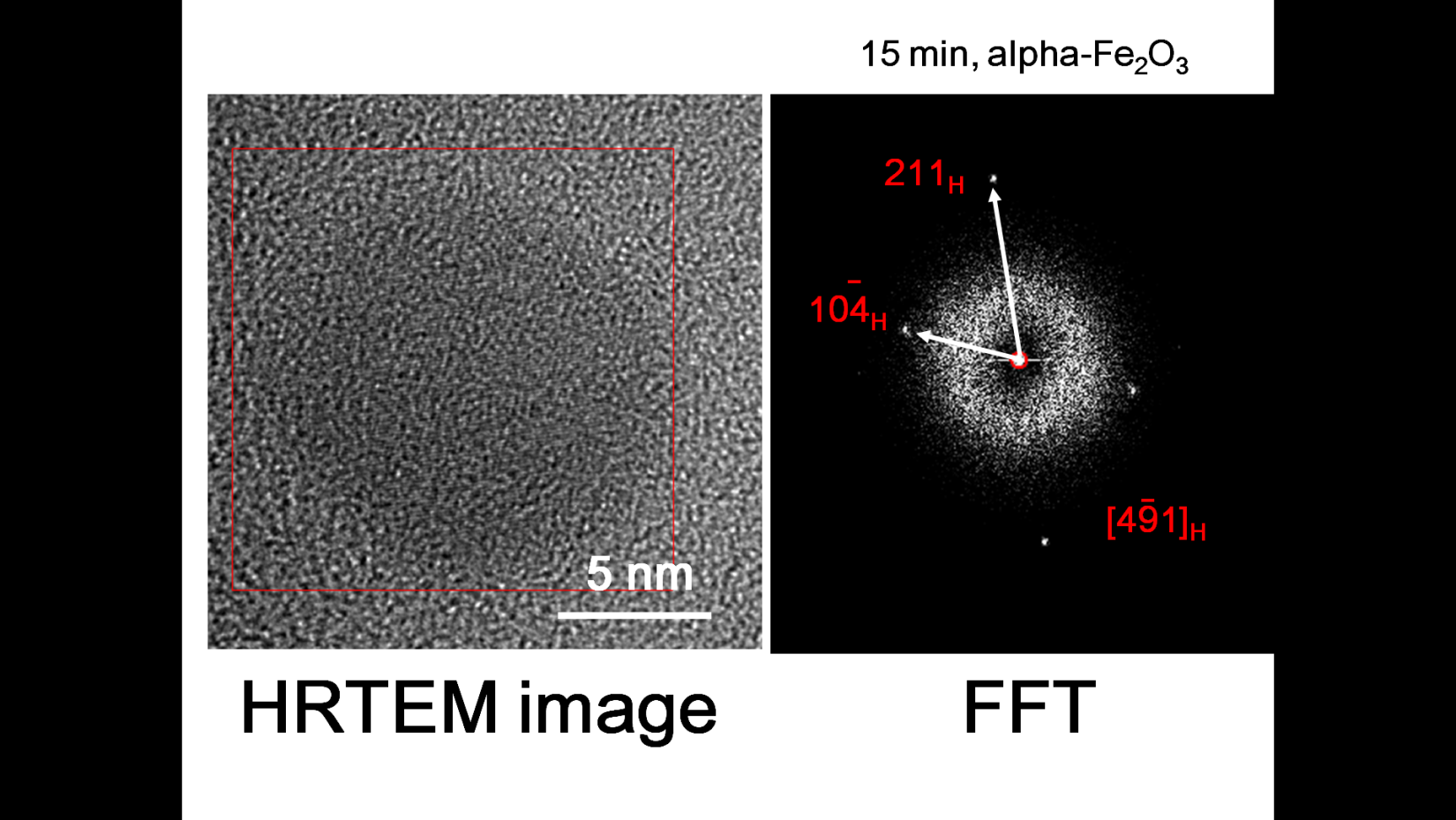

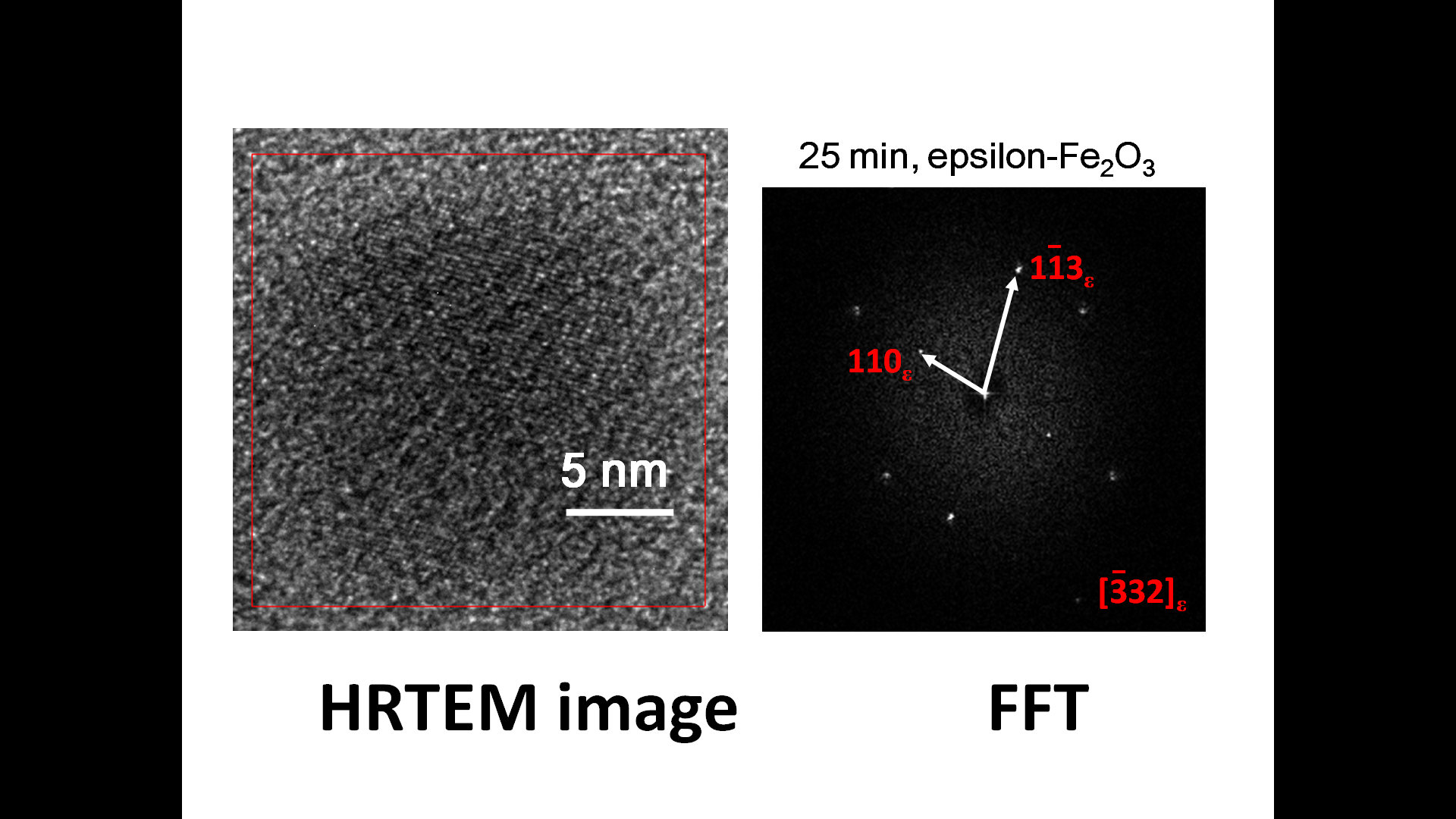


**Supplementary Information Table S1**. Crystallographic information of the *ε*-Fe_2_O_3_ exhibited in Figure 3a, Figure 4 and the theoretical data of *ε*-Fe_2_O_3_ and Fe_3_O_4_ (magnetite). For further crystallographic information, people can refer to pdf card as 33-0664 for *α*-Fe_2_O_3_, 65-3107 for magnetite and 52-1449 for *ε*-Fe_2_O_3_, respectively.

| Image identifier | 5 min | 10 min | 15 min | 25 min |
| --- | --- | --- | --- | --- |
|  |  |  |  |  |
| Axis of projection, as indicated in Fig. 2 for epsilon-Fe_2_O_3_ | [-332] | [-332] | [-10 4 1] | [10 -8 3] |
| Reflections identified for epsilon-Fe_2_O_3_ | 110 and 1-13 | 110 and 1-13 | 122 and 13-2 | 21-4 and 122 |
|  |  |  |  |  |
| Possible axis of projection for magnetite | [03-1] | [03-1] | [4 1 -1] | [4 1 -1] |
| Possible reflections identified for magnetite | 200* and 113 | 200* and 113 | 022 and 1-13 | 1-31 and 115 |
|  |  |  |  |  |
| Ratio measured between two sets of reciprocal spacings (referred to as r1 and r2) | 1.68  r1 = 4.36 Å and r2 = 2.59 Å | 1.65  r1 = 4.31 Å and r2 = 2.61 Å | 1.2  r1 = 2.73 Å and r2 = 2.28 Å | 1.61  r1 = 2.68 Å and r2 = 1.66 Å |
| Ratio between spacings as indicated in Fig. 2 for epsilon-Fe_2_O_3_ (referred to as r1 and r2) | 1.72  r1 = 4.39 Å and r2 = 2.55 Å | 1.72  r1 = 4.39 Å and r2 = 2.55 Å | 1.22  r1 = 2.71 Å and r2 = 2.23 Å | 1.6  r1 = 2.71 Å and r2 = 1.69 Å |
| Ratio between spacings in magnetite (referred to as r1 and r2) | 1.66  r1 = 4.2 Å and r2 = 2.53 Å | 1.66  r1 = 4.2 Å and r2 = 2.53 Å | 1.17  r1 = 2.97 Å and r2 = 2.53 Å | 1.57  r1 = 2.53 Å and r2 = 1.61 Å |
|  |  |  |  |  |
| Angle measured between two sets of reciprocal spacings (degrees) | 71 | 70.5 | 64.7 | 85 |
| Angle between reciprocal lattice directions as indicated in Fig. 2  for epsilon-Fe_2_O_3_ | 73 | 73 | 64.1 | 86.4 |
| Angle between reciprocal lattice directions in magnetite | 72.5 | 72.5 | 64.8 | 80 |
